# Topological and functional comparison of community detection algorithms in biological networks

**DOI:** 10.1186/s12859-019-2746-0

**Published:** 2019-04-27

**Authors:** Sara Rahiminejad, Mano R. Maurya, Shankar Subramaniam

**Affiliations:** 1 Departments of Bioengineering and Mechanical and Aerospace Engineering, University of California, San Diego, 9500 Gilman Dr, La Jolla, CA 92093 USA; 2 Department of Bioengineering and San Diego Supercomputer Center, University of California, San Diego, 9500 Gilman Dr, La Jolla, CA 92093 USA; 3 Department of Bioengineering, Departments of Computer Science and Engineering, Cellular and Molecular Medicine, and the Graduate Program in Bioinformatics, University of California, San Diego, 9500 Gilman Dr, La Jolla, CA 92093 USA

**Keywords:** Biological networks, Community detection, Modularity, Biological function, Pathways

## Abstract

**Background:**

Community detection algorithms are fundamental tools to uncover important features in networks. There are several studies focused on social networks but only a few deal with biological networks. Directly or indirectly, most of the methods maximize modularity, a measure of the density of links within communities as compared to links between communities.

**Results:**

Here we analyze six different community detection algorithms, namely, Combo, Conclude, Fast Greedy, Leading Eigen, Louvain and Spinglass, on two important biological networks to find their communities and evaluate the results in terms of topological and functional features through Kyoto Encyclopedia of Genes and Genomes pathway and Gene Ontology term enrichment analysis. At a high level, the main assessment criteria are 1) appropriate community size (neither too small nor too large), 2) representation within the community of only one or two broad biological functions, 3) most genes from the network belonging to a pathway should also belong to only one or two communities, and 4) performance speed. The first network in this study is a network of Protein-Protein Interactions (PPI) in *Saccharomyces cerevisiae* (Yeast) with 6532 nodes and 229,696 edges and the second is a network of PPI in *Homo sapiens* (Human) with 20,644 nodes and 241,008 edges. All six methods perform well, i.e., find reasonably sized and biologically interpretable communities, for the Yeast PPI network but the Conclude method does not find reasonably sized communities for the Human PPI network. Louvain method maximizes modularity by using an agglomerative approach, and is the fastest method for community detection. For the Yeast PPI network, the results of Spinglass method are most similar to the results of Louvain method with regard to the size of communities and core pathways they identify, whereas for the Human PPI network, Combo and Spinglass methods yield the most similar results, with Louvain being the next closest.

**Conclusions:**

For Yeast and Human PPI networks, Louvain method is likely the best method to find communities in terms of detecting known core pathways in a reasonable time.

**Electronic supplementary material:**

The online version of this article (10.1186/s12859-019-2746-0) contains supplementary material, which is available to authorized users.

## Background

The use of networks to study complex interacting systems has been applied to many domains during the last two decades, including sociology, physics, computer science and biology. An important task in the analysis of networks lies in the identification of communities or modules whose membership share one or more common features of the system. The problem that community detection attempts to solve is the identification of groups of nodes with more and/or better interactions amongst its members than between its members and the remainder of the network [1, 2]. For example, in social networks, a community may correspond to groups of friends who attend the same school or live in the same neighborhood; while in a biological network, communities may represent functional modules of interacting proteins.

Edges in a biological network may represent various types of direct interactions and indirect effects. Examples of direct interactions include protein-protein interactions as part of signaling pathways or as part of protein complexes and substrate-enzyme interactions. Indirect effects may include transport processes and regulatory effects, which, in most cases, can be substituted with a subnetwork of several direct interactions when modeled at a finer granularity. Examples of the latter are cholesterol and ion transport across the plasma membrane and protein-DNA interactions in gene-regulatory networks. Thus, in the context of a cell or tissue, subnetworks or communities may correspond to various cellular processes, pathways and functions, in which its components (nodes) exhibit a higher-degree of interaction as compared to those from outside the pathway.

Majority of the methods for community detection in networks are based on maximization of modularity. While the modularity metric Q, of a network, is defined in the Methods section, intuitively, given a network, if it can be partitioned in such a way that only a few connections exist between the nodes of different partitions and most connections are among the nodes within the partitions, then the modularity will be high. It is interesting to note that the modularity of a sparse network of fully connected subnetworks is higher than that of a fully connected network, which is zero. Any partition of a fully connected network results in Q < 0. Brandes et al. have carried out extensive theoretical analysis of properties of modularity and complexity of its maximization [3].

One of the most important objectives of any large-scale omics study is to identify mechanisms for specific functions and phenotypes in a chosen context. Biological networks derived from genome-scale experimental data and/or legacy knowledge are generally large and complex with thousands of nodes and many thousands of connections. Associating meaningful biological functions and interpretations to such networks is impossible. However, these large networks can be broken down into smaller (sub) networks (also called as modules or communities) which are more amenable to biological interpretation. Such communities are expected to represent one or a few biological functions and they may facilitate discovery of mechanisms relating the causes or perturbations to the observed phenotypes. Thus, community detection can provide valuable biological insights.

Several methods have been developed to find communities in networks using tools and techniques from different disciplines such as applied mathematics or statistical physics [4]. All these methods try to identify meaningful communities, while keeping the computational complexity of the underlying algorithm low [5]. Although these methods have proven to be successful in some cases, there is no guarantee that the resulting communities provide the best functional description of the system. Hence, selecting a suitable method to detect communities in a network is challenging. While there have been some studies comparing different methods for community detection [5], their focus has been on Lancichinetti, Fortunato, Radicchi (LFR) benchmark networks (artificial networks that have heterogeneity in the distributions of degree of nodes and the size of communities) [6]; comparisons with respect to biological networks are lacking.

Classical community detection algorithms initially divide networks into communities according to some network features such as edge betweenness. One of the most popular and prominent algorithms that uses edge betweenness is the Girvan-Newman algorithm [1, 7]. In this method edges are progressively removed from the original network till the modularity reaches its maximum value, making it an optimization problem. The connected nodes of the remaining network are the communities. The Girvan-Newman algorithm has been successfully applied to a variety of networks, including networks of email messages. However, its computational complexity, *O(m*^*2*^*n)* for a network with *n* nodes and *m* edges, practically restricts its use to networks of at most a few thousand nodes. There are other optimization-based algorithms with different objective functions that provide different approaches to solve the community detection problem. For example, Leading Eigen [8] algorithm also tries to maximize modularity but the modularity is expressed in the form of the eigenvalues and eigenvectors of a matrix called the modularity matrix. Spinglass method minimizes the Hamiltonian of the network [9].

Since the early 2000s, several methods have been developed that divide networks into communities based on the modularity [10–15]. The modularity criterion was revisited in 2005 when Duch and Arenas proposed a divisive algorithm [16] that optimizes the modularity using a heuristic search based on the Extremal Optimization (EO) algorithm proposed by Boettcher and Percus [17, 18]. Pizzuti has suggested an algorithm named *GA-net* that uses a special assessment function described as the community score in addition to the modularity function [19]. There are also other approaches to the community detection problem in which the use of multiple objectives (or assessment criteria) is preferred over the use of a single objective for complex networks. Since the objectives are usually directly related to the network properties, one advantage of using multi-objective optimization is that it balances among the multiple (important) properties of the network. The benefits of using multi-objective approach have been explained by Shi et al. [20].

In this manuscript, we briefly review eight algorithms for finding communities in biological networks such as Protein-Protein Interaction (PPI) networks (discussed in the Methods section). In such networks, each node represents a protein (or gene) and each edge represents an interaction between two proteins. In particular, we will apply six algorithms to the Yeast PPI network with 6532 nodes and 229,696 edges and the Human PPI network with 20,644 nodes and 241,008 edges. Using several topological metrics, we assess which methods provide similar (or dissimilar) results. We evaluate the biological interpretation of the communities identified and compare the results in terms of their functional features. At a high level, the main criteria for assessment of the methods are 1) appropriate community size (neither too small nor too large), 2) representation within the community of only one or two broad biological functions, 3) most genes from the network belonging to a pathway should also belong to only one or two communities, and 4) performance speed.

This paper is organized as follows: in the next section we will present the results of applying six methods on the Yeast and Human PPI networks and compare the communities based on their topological and functional features. In the last part of this section, we will describe an orthology analysis between the communities detected for the Yeast PPI network and the communities detected for the Human PPI network. In the following section, we will present discussion on the results providing insights into the algorithmic similarities and robustness of some of the methods. In the section after that, we will provide the conclusion of our paper. In the Methods section, we will describe eight different methods for finding communities in networks. We will also introduce three metrics to compare the communities identified by the algorithms.

## Results

Six community detection methods, namely, Combo, Conclude, Fast Greedy, Leading Eigen, Louvain and Spinglass, have been applied to the Yeast PPI network with 6532 nodes and 229,696 edges and the Human PPI network with 20,644 nodes and 241,008 edges. A detailed description of the methods is included in the Methods section. We used the BioGRID database [21, 22] for the PPI networks for Yeast and Human. Since our focus in this paper is on undirected and unweighted networks, we removed repeated edges and self-loops from our data set.

In the first part of this section, we will present the results for the Yeast PPI network. In the second part, the results for the Human PPI network will be presented. In the third part, an orthology comparison will be provided between the Yeast and Human PPI networks.

### Yeast PPI network

Among the methods tested to find communities of the Yeast PPI network, Combo, Conclude, Fast Greedy, Leading Eigen, Louvain and Spinglass give good partitioning results, i.e., the size of communities detected are not too small or too large compared to the size of the original network. Since the Yeast PPI network has 6532 nodes, Girvan-Newman algorithm is not an appropriate method to detect communities. It takes 44 min (on a PC with 4 GB RAM with 4 2.4 GHz processors) for Rattus PPI network which has 3379 nodes and 4580 edges. Its computational complexity is proportional to *m*^*2*^*n* (where *n* is the number of nodes and *m* is the number of edges), so, it will take ~ 148 days to find communities in the Yeast PPI network (using the computational resource mentioned above). Infomap, is also not a good method based on the size of communities it detects; the largest community has 6195 nodes and the smallest one has just 2 nodes. Since very small communities (e.g., those with less than 100 nodes) are not expected to yield significant biological insights, we will not consider them in our analysis. We note that there may be some exceptions.

In the next subsection, first we will compare the methods from a topological perspective of the communities identified. Then we will provide a functional comparison. To begin with, the results for all these methods are described in Table 1 in terms of the size of the communities detected for the Yeast PPI network.

**Table 1:** Number of nodes and edges for communities detected using different methods for the Yeast PPI network (6532 nodes and 229,696 edges). The number in parenthesis after the name of each method represents the number of communities detected by that method. For example, Combo finds 8 communities. Modularity scores are also provided for different methods. For each method, we only consider the communities with 100 or more nodes and list up to 10 communities

Combo (8) Q = 0.2654	# of nodes	2231	1514	1337	1284						
	# of edges	25,137	23,690	38,523	30,585						
Conclude (66) Q = 0.2468	# of nodes	788	744	602	468	423	359	288	271	252	199
	# of edges	14,585	10,506	3272	988	5826	4123	1404	3486	940	1703
F. Greedy (10) Q = 0.2112	# of nodes	2608	2410	1466							
	# of edges	61,665	72,998	7180							
L. Eigen (4) Q = 0.1686	# of nodes	2661	1910	984	977						
	# of edges	75,812	28,664	7373	7203						
Louvain (9) Q = 0.2643	# of nodes	1538	1472	1190	1151	993	131				
	# of edges	16,015	23,394	13,247	31,202	22,553	676				
Spinglass (9) Q = 0.2681	# of nodes	1607	1473	1194	1148	1076					
	# of edges	16,616	23,876	12,282	32,641	23,854					

### Comparison based on topological features of communities

The following table (Table 1) represents the results for applying six methods on the Yeast PPI network.

Using three different metrics, namely, Rand Index (RI), Adjusted Rand Index (ARI), and Normalized Mutual Information (NMI) (described in the Methods section), we are able to compare different pair of methods. Table 2 represents the results of comparing six methods (Combo, Conclude, Fast Greedy, Leading Eigen, Louvain and Spinglass) with respect to three topological metrics (RI, ARI and NMI).

**Table 2:** Comparison of different methods with respect to three topological metrics, namely, Rand Index (*RI*), Adjusted Rand Index (*ARI*) and Normalized Mutual Information (*NMI*) for the Yeast PPI network. When a method is compared with itself, RI, ARI and NMI are 1 (diagonal elements). Larger (smaller) the value of RI, ARI and NMI, the more (less) similar are the two methods being compared. For example, Louvain and Spinglass are most similar to each other

		Combo	Conclude	F. Greedy	L. Eigen	Louvain	Spinglass
RI	Combo	1	0.7608	0.7157	0.6788	0.8319	0.8409
ARI	Combo	1	0.1466	0.3125	0.1942	0.5163	0.5479
NMI	Combo	1	0.2905	0.4024	0.2447	0.5413	0.5723
RI	Conclude		1	0.6815	0.7061	0.8083	0.8012
ARI	Conclude		1	0.0818	0.0825	0.1659	0.1637
NMI	Conclude		1	0.1956	0.1472	0.3016	0.2924
RI	F. Greedy			1	0.6334	0.7098	0.7129
ARI	F. Greedy			1	0.146	0.2629	0.2764
NMI	F. Greedy			1	0.1918	0.3545	0.3652
RI	L. Eigen				1	0.6952	0.6936
ARI	L. Eigen				1	0.188	0.1914
NMI	L. Eigen				1	0.2231	0.2285
RI	Louvain					1	0.9021
ARI	Louvain					1	0.6922
NMI	Louvain					1	0.6644
RI	Spinglass						1
ARI	Spinglass						1
NMI	Spinglass						1

Based on the results of Table 2, Louvain and Spinglass are most similar to each other amongst all pairs of comparisons. To maintain consistency in finding dissimilar methods, we selected a method which is dissimilar to Louvain, e.g., Conclude or Leading Eigen. Since Conclude finds 66 communities with sizes (number of nodes) ranging from 3 to 788, we compare Louvain with Leading Eigen here. We present the results from comparing Louvain and Conclude in the Additional file 1.

Table 3 provides Jaccard index (as a percentage) between communities identified by Louvain and Spinglass. We used *Intersect* function in R to find common genes between two communities and then divided the number of common genes by the total number of unique genes between the two communities (*union* function in R) to get the Jaccard index. Table 4 uses the same approach to find Jaccard index for communities detected by dissimilar methods, in particular, Louvain and Leading Eigen. The rest of Jaccard index matrices amongst all pairs of communities for all methods can be found in the Additional file 1: Table S1.

**Table 3:**
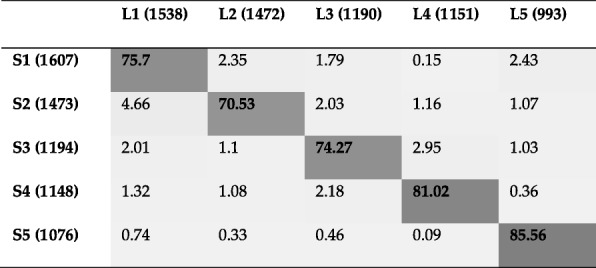
Jaccard index (as a percentage) between the communities identified by two similar methods, namely, Louvain and Spinglass, for the Yeast PPI network. L1 to L5 refer to the communities detected by Louvain method and sorted by their size. Similarly, S1 to S5 refer to the communities detected by Spinglass method. The numbers in parenthesis represent the number of genes in each community. Community pairs with maximum overlap (e.g., L1 vs. S1) are indicated in bold text

**Table 4:**
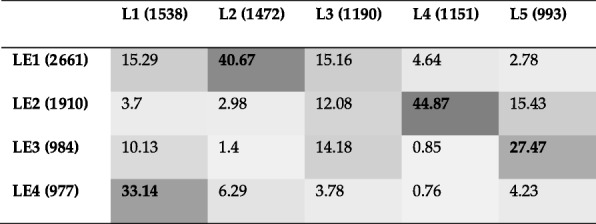
Jaccard index (as a percentage) between the communities identified by two dissimilar methods, namely, Louvain and Leading Eigen for the Yeast PPI network. (L1 to L5: communities detected by Louvain; LE1 to LE4: communities detected by Leading Eigen). The numbers in parenthesis represent the number of genes in each community. Community pairs with maximum overlap (e.g., L1 vs. LE4) are indicated in bold text

### Comparison based on biological/functional features of communities

As described in the previous subsection, Louvain and Spinglass are most similar to each other and Louvain and Leading Eigen are most dissimilar. In order to know which communities of similar and dissimilar methods have to be compared to each other, we analyzed Tables 3 and 4 (Jaccard index) for all pairs of communities between similar and dissimilar methods. After selecting pairs of communities with highest value of Jaccard index for each column, we used Database for Annotation, Visualization and Integrated Discovery (DAVID) version 6.8 [23, 24] to perform Kyoto Encyclopedia of Genes and Genomes (KEGG) [25] pathway and Gene Ontology (GO) term (GOTERM_BP_3) enrichment analysis for each community. In the following Tables (Table 5 through Table 7), we have considered pathways with more than 10 genes and with *p*-values less than or equal to 0.01. The number in parenthesis (e.g., after L1 or S1) is the number of genes that DAVID could annotate for that specific community for the Yeast PPI network. For example, the first community of Louvain (L1) has 1538 genes (Table 1) and of those, DAVID is able to annotate 1481 genes. In Tables 5, 6, 7, the first column lists the broad category of pathways (M: Metabolism, CP: Cellular Processes, GIP: Genetic Information Processing, HD: Human Diseases, and OS: Organismal Systems). The second column lists the different pathways enriched. Columns 3 and 7 (Count) represent number of genes enriched in the pathways, columns 4 and 8 (*p*-value) represent *p*-values for those pathways in the communities compared, and columns 5 and 9 (FE) represent Fold Enrichment for the pathways. FE is defined as (s/b)/(k/N) where b is the total number of genes in a chosen pathway; s, the number of genes from the community in this pathway; N, the total number of genes for the species; and k, the number of genes in the community; all the four numbers are based on intersection/overlap with the respective DAVID database (e.g., KEGG). Essentially, FE represents the relative increase or decrease of the fraction of genes from the set of interest belonging to a pathway as compared to the genes from a background set (generally covering the whole-genome) belonging to the same pathway. The values in columns 5 and 9 are shaded light to dark with increasing FE. Column 6 (common) is the number of genes common to both communities for the different pathways.

**Table 5:**
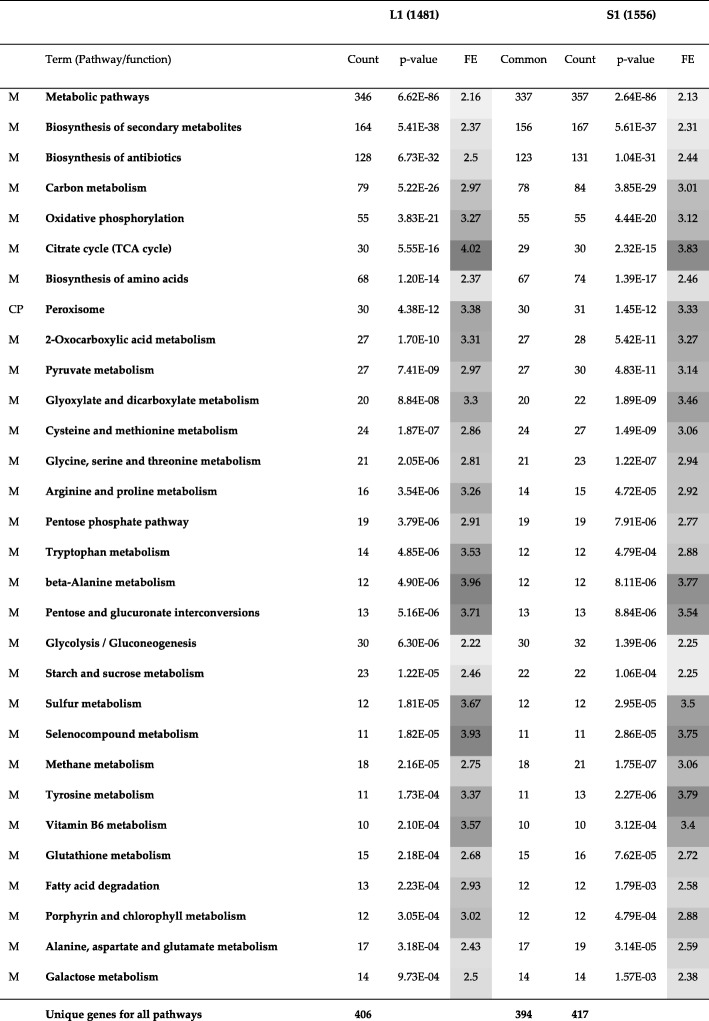
A Comparison of KEGG pathway enrichment results between the first community of Louvain (L1) with 1538 genes and the first community of Spinglass (S1) with 1607 genes for the Yeast PPI network. The numbers inside parenthesis after L1 and S1 represent the number of genes that DAVID could annotate, which is generally less than the number of genes in those communities. The first column lists the broad category of pathways (*M*: Metabolism, *CP*: Cellular Processes). Many pathways enriched in L1 and S1 have good overlap (a large number of genes are common). *FE*: Fold Enrichment. False Discovery Rate (*FDR*) values for all pathways and both communities are approximately 1.10E+3 times p-value (the factor 1.10E+3 is related to the size of the community)

**Table 6:**
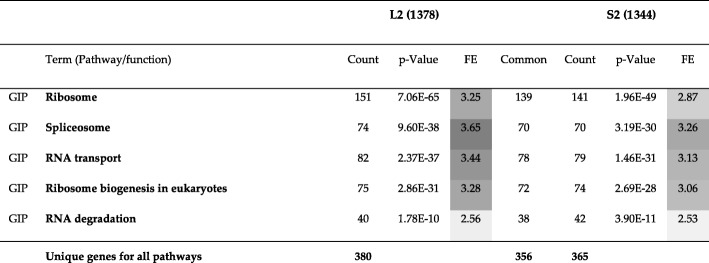
A comparison of KEGG pathway enrichment results between the second community of Louvain (L2) with 1472 genes and the second community of Spinglass (S2) with 1473 genes for the Yeast PPI network. The numbers inside parenthesis after L2 and S2 represent the number of genes that DAVID could annotate, which is generally less than the number of genes in those communities. The first column lists the broad category of pathways (*GIP*: Genetic Information Processing). Many pathways enriched in L2 and S2 have good overlap (a large number of genes are common). *FE*: Fold Enrichment. False Discovery Rate (*FDR*) values for all pathways and both communities are approximately 1.05E+3 times *p*-value

**Table 7:**
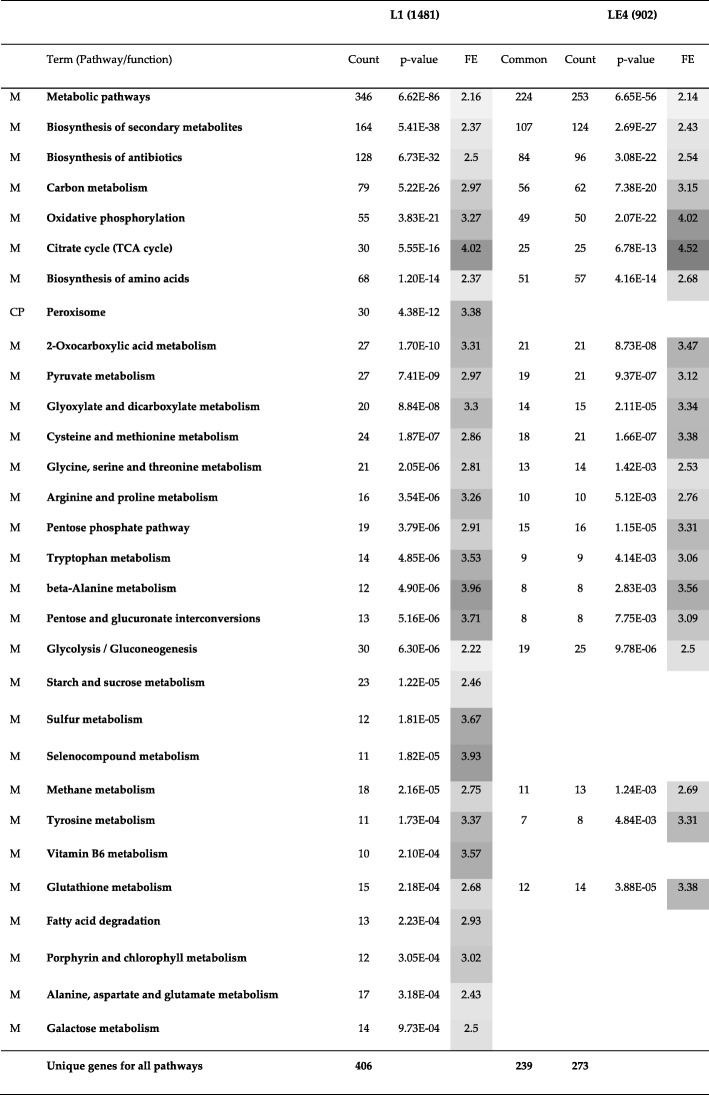
A comparison of KEGG pathway enrichment results between the first community of Louvain with 1538 genes and the fourth community of Leading Eigen (LE4) with 977 genes for the Yeast PPI network. The numbers inside parenthesis after L1 and LE4 represent the number of genes that DAVID could annotate, which is generally less than the number of genes in those communities. The first column lists the broad category of pathways (*M*: Metabolism, and *CP*: Cellular Processes), *FE*: Fold Enrichment, False Discovery Rate (*FDR*) values for all pathways and both communities are approximately 1.10E+3 times p-value

#### Comparing similar methods

As the first column of Table 3 shows, the first community of Louvain (L1) and the first community of Spinglass (S1) have the maximum overlap and the results of comparing KEGG pathway enrichment analysis between L1 and S1 are presented in Table 5. Additional file 1: Table S2 shows the results of comparing GO term enrichment analysis between these two communities. L2 and S2 are 71% similar to each other based on Table 3 and they are compared in Table 6 for KEGG pathway enrichment analysis and in Additional file 1: Table S3 for GO term enrichment analysis. The rest of the comparison Tables (KEGG pathway enrichments analysis for L3 vs. S3, L4 vs. S4, and L5 vs. S5) are in the Additional file 1: Tables S4-S6. Since DAVID did not find any pathways for small communities, such as L6 which has 131 nodes, those communities are not considered in the comparison Tables.

Based on Table 3, L1 and S1 are 76% similar to each other in terms of Jaccard index. In Table 5, KEGG pathway enrichment results of these two communities reveal that majority of these genes are related to various metabolic pathways such as carbohydrate metabolism, energy metabolism, amino acid metabolism and metabolism of cofactors and vitamins. The top four pathways represent broad metabolism pathways. There are 13 pathways categorized as amino acid metabolism such as cysteine and methionine metabolism, or glycine, serine and threonine metabolism. Among pathways that are categorized as energy metabolism, oxidative phosphorylation is the one with the lowest *p*-value.

In terms of enzyme commission annotation, there are 1738 enzyme-coding genes in the entire network. L1 and S1 have 571 and 605 enzyme-coding genes, respectively. Of these, 529 enzyme-coding genes are common between the two communities, which shows a significant overlap. There are a few enzyme-coding genes which are present in L1 but not in S1 such as aminoacyl-tRNA hydrolase (PTH1) or glutamate 5-kinase (PRO1). Similarly, for genes that are present in S1 but not in L1, sulfuric ester hydrolase (BDS1) is an example. Since both Louvain and Spinglass find 9 non-overlapping communities, all enzyme-coding genes are part of one of the communities.

Table 6 shows KEGG pathway enrichment results for the communities L2 and S2. All pathways are related to genetic information processing with approximately similar genes enriched in the two methods. The first pathway with the lowest *p*-value is ribosome which is a complex molecule made of ribosomal RNA molecules and proteins. There are 151 genes enriched in L2 and 141 genes enriched in S2 for this pathway. Of these, 139 genes are common (a 92% overlap). Similar trend is observed for other pathways as well, e.g., there is a 95% overlap between L2 and S2 for Spliceosome and 95% overlap for RNA transport.

The GO term enrichment results shown in Additional file 1: Tables S2 and S3 also verify the similarity between L1 and S1, and L2 and S2, respectively. Counting all genes for all pathways in Additional file 1: Table S2 yields 1062 unique genes for L1 and 1103 unique genes for S1 and of these, 957 genes are common between the two communities, which is an 87% overlap. This similarity value is 84% between L2 and S2 (Additional file 1: Table S3).

Additional file 1: Table S4 provides a comparison between the communities L3 and S3. The pathways enriched can be classified into four different groups (metabolic processes, environmental information processing, cellular processes and human diseases) as opposed to just one or two. Still, the overlap between L3 and S3 communities for each of the pathways is more than 80%.

L4 and S4 are 81% similar to each other based on Table 3 and the results of their comparison are shown in Additional file 1: Table S5. Most pathways for these two communities are related to genetic information processing category. There are two pathways related to cellular processes and two pathways related to metabolic processes. Based on KEGG pathway enrichment results, there is a good overlap between genes enriched in different pathways for these two communities. For example, 71 genes of L4 are enriched in cell cycle pathway and 77 genes of S4 are also enriched in this pathway. Among genes enriched in cell cycle pathway, 70 genes are common between L4 and S4, giving a 91% overlap.

Additional file 1: Table S6 compares L5 and S5. Based on Table 3, they are 86% similar to each other. KEGG pathway enrichment results of these two communities show that most pathways are related to metabolic processes and there are also other pathways related to other categories such as endocytosis, which is in the cellular processes category. The results of KEGG pathway also verify the similarity of Table 3. As seen from the enriched pathways, almost all of them have the same genes enriched in both communities. For example, there are 29 genes for L5 enriched in N-Glycan biosynthesis and the same genes are found in S5 in the same pathway.

#### Comparing dissimilar methods

In this subsection we will compare the methods that are most dissimilar to each other, namely Louvain and Leading Eigen. As Table 4 shows, the first community of Louvain has the maximum overlap with the fourth community of Leading Eigen (LE4). The results of this comparison based on KEGG and GO term enrichment analysis are shown in Tables 7 and Additional file 1: Table S7, respectively. The rest of the comparisons can be found in the Additional file 1: Table S8 for L2 vs. LE1, Additional file 1: Table S9 for L4 vs. LE2 and Additional file 1: Table S10 for L5 vs. LE3).

The first pathway of Table 7 with the lowest *p*-value is metabolic pathways with 346 genes enriched in L1 and 253 genes enriched in LE4. Of these genes, 224 genes are common between the two communities which is a 65% overlap. In contrast, the first pathway of Table 5 shows that 337 genes are common between L1 and S1, which is a 94% overlap. There are some pathways in Table 7 that are blank for LE4 such as biosynthesis of amino acids. For these pathways, although there are some genes enriched in L1, there are no genes enriched in LE4 or if there are any, the *p*-value for the pathway is higher than the defined cut-off of 0.01. Counting all genes for all pathways yields 406 unique genes for L1 and 273 unique genes for LE4 and of these, 239 genes are common between the two communities which is a 59% overlap. Based on GO term enrichment analysis shown in Additional file 1: Table S7, there are 474 genes common between L1 and LE4 out of 1062 unique genes for L1 and 662 unique genes for LE4, which is a 45% overlap. These relatively low best-overlaps also confirm that these two methods are dissimilar to each other.

Based on Table 2, Louvain and Conclude methods are also dissimilar to each other. We compared the communities obtained from these two methods. As Additional file 1: Table S1 shows, the first community of Louvain (L1) has the maximum overlap with the third community of Conclude (CL3). The results of this comparison based on KEGG pathway enrichment analysis are shown in Additional file 1: Table S11. The metabolic pathways is the most enriched pathway with 346 genes enriched in L1, 230 genes enriched in CL3, and 173 genes common between the two communities, which is a 50% overlap. Counting all unique genes for all pathways yields 406 genes for L1 and 241 genes for CL3 and of these, 181 genes are common between the two communities (which is a 45% overlap). This similarity value is close to what we calculated for Louvain vs. Leading Eigen, using KEGG pathway and GO term enrichment analysis (Table 7 and Additional file 1: Table S7). The rest of the comparisons can be found in Additional file 1: Table S12 for L2 vs. CL2, Additional file 1: Table S13 for L4 vs. CL1, and Additional file 1: Table S14 for L5 vs. CL5. Overall, dissimilarity at the topological level translates into dissimilarity at the functional level as well.

### Human PPI network

Six methods, namely, Combo, Conclude, Fast Greedy, Leading Eigen, Louvain, and Spinglass, have been applied to the Human PPI network with 20,644 nodes and 241,008 edges. Although all of them were able to find communities, we will not consider the results of Conclude because it finds 495 communities, many of which are very small communities with less than 50 nodes. For Combo and Spinglass, since they use a random number generator in the procedure of finding communities, we ran them 10 times with 10 different seeds between 0 and 10,000 and used the results from the run with the largest modularity. Modularity scores and the number of communities detected in each run for Combo and Spinglass are summarized in Table 8 and Table 9, respectively.

**Table 8:** Modularity scores and number of communities detected by Combo for the Human PPI network. Each run uses a random seed between 0 and 10,000 in the procedure for finding communities

Modularity	0.3735	0.3734	0.3734	0.3729	0.3723
# of communities detected	11	13	11	15	11
Modularity	0.3723	0.3718	0.3715	0.3711	0.3704
# of communities detected	13	12	10	12	13

**Table 9:** Modularity scores and number of communities detected by Spinglass for the Human PPI network. Each run uses a random seed between 0 and 10,000 in the procedure for finding communities

Modularity	0.3729	0.3727	0.3725	0.3725	0.3724
# of communities detected	21	22	22	24	21
Modularity	0.3721	0.3716	0.3716	0.3711	0.3688
# of communities detected	22	23	21	23	23

After finding modularity scores and communities for 10 runs, we selected communities corresponding to the largest modularity score which is 0.3735 for Combo (11 communities) and 0.3729 (21 communities) for Spinglass.

The results of comparing all methods excluding Conclude are presented in Table 10.

**Table 10:** Comparison of different methods with respect to three topological metrics, namely, RI, ARI and NMI for the Human PPI network (20,644 nodes and 241,008 edges). When a method is compared with itself, RI, ARI and NMI are 1 (diagonal elements). Larger (smaller) the value of RI, ARI and NMI, the more (less) similar are the two methods being compared. For example, Combo and Spinglass are most similar to each other, Louvain being the next most similar to them. Overall, Combo, Louvain and Spinglass provide similar results

		Combo	F. Greedy	L. Eigen	Louvain	Spinglass
RI	Combo	1	0.7314	0.3606	0.8805	0.8948
ARI	Combo	1	0.1806	0.0315	0.416	0.4998
NMI	Combo	1	0.3025	0.0936	0.4601	0.5551
RI	F. Greedy		1	0.444	0.7243	0.7258
ARI	F. Greedy		1	0.0624	0.1609	0.1739
NMI	F. Greedy		1	0.0787	0.2682	0.3063
RI	L. Eigen			1	0.3531	0.3649
ARI	L. Eigen			1	0.0191	0.0326
NMI	L. Eigen			1	0.0711	0.0951
RI	Louvain				1	0.8832
ARI	Louvain				1	0.4479
NMI	Louvain				1	0.4679
RI	Spinglass					1
ARI	Spinglass					1
NMI	Spinglass					1

**Fig. 1:**
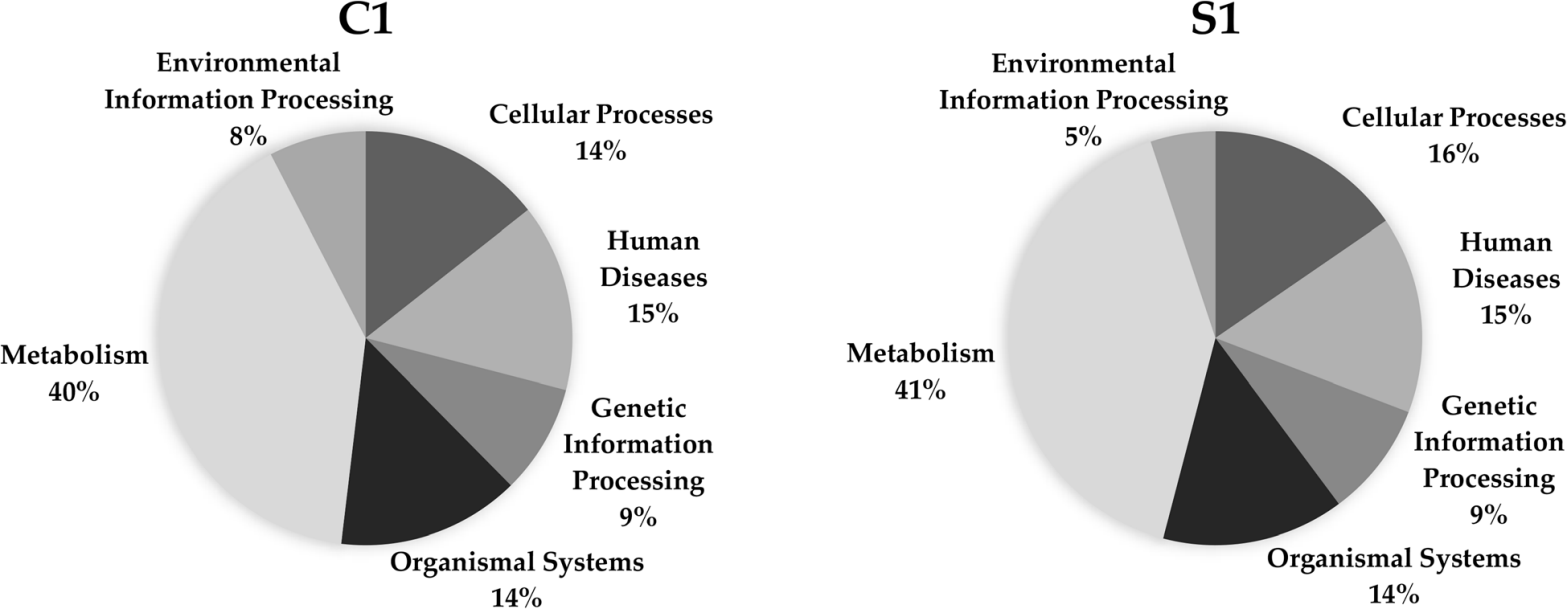
Pie charts for KEGG pathway enrichment results of C1 with 3252 genes and S1 with 3206 genes for the Human PPI network. Left chart shows the results for C1 and right chart shows the results for S1

**Fig. 2:**
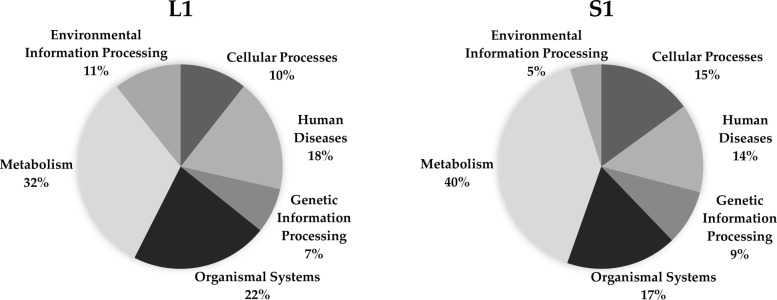
Pie charts for KEGG pathway enrichment results of L1 with 3585 genes and S1 with 3206 genes for the Human PPI network. Left chart shows the results for L1 and right chart shows the results for S1

From Table 10, we can see that Louvain and Spinglass are more similar to each other as compared to all other pairs of methods except Combo and Spinglass. Hence, we will compare Combo and Spinglass as well here. Since they are more similar to each other than Louvain and Spinglass, they will be compared first. The first community of Combo (C1) and the first community of Spinglass (S1) have been compared to each other using KEGG pathway enrichment analysis and the results for top 10 pathways are presented in Table 11. Table 12 presents the results of comparing top 10 pathways for the first community of Louvain (L1) and the first community of Spinglass (S1). Organization of these two tables is the same as that for Tables 5, 6 and 7 in the previous subsection. The complete versions of Tables 11 and 12 are in the Additional file 1: Tables S15 and S16, respectively. The results of comparing all pathways for the communities C1 and S1 and L1 and S1 (with *p*-values less than 0.01) are illustrated in Fig. 1 and Fig. 2, respectively. The pie charts in Figs. 1 and 2 show the broad functional categories. Essentially, pathways belonging to a broad category are selected and the genes of these pathways combined together and the number of unique genes is expressed as a percentage of total unique genes in all pathways with p-values less than 0.01. As an example, there are three different pathways belong to cellular processes in C1: lysosome, peroxisome and phagosome. There are 61 genes enriched in lysosome, 46 genes enriched in peroxisome and 63 genes enriched in phagosome. Together, they have 159 unique genes, which is about 14% of the total unique genes for all pathways with p-value less than 0.01. We performed these calculations for all six broad categories of pathways for two community-pairs of Additional file 1: Tables S15 and S16 and the corresponding results are shown in Figs. 1 and 2, respectively.

**Table 11:**
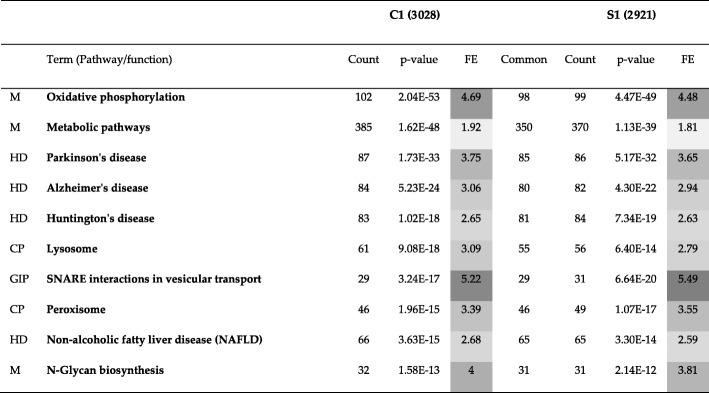
Top 10 pathways for a comparison of KEGG pathway enrichment results between C1 with 3252 genes and S1 with 3206 genes for the Human PPI network (20,644 nodes and 241,008 edges). The numbers inside parenthesis after C1 and S1 represent the number of genes that DAVID could annotate, which is generally less than the number of genes in those communities. The first column lists the broad category of pathways (*M*: Metabolism, *HD*: Human Diseases, *CP*: Cellular Processes, and *GIP*: Genetic Information Processing), *FE*: Fold Enrichment

**Table 12:**
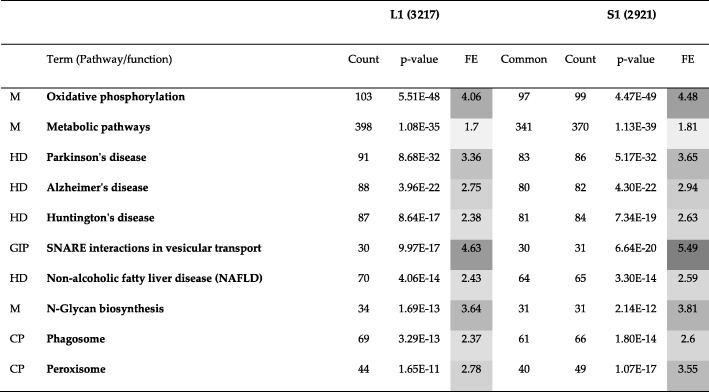
Top 10 pathways for a comparison of KEGG pathway enrichment results between L1 with 3585 genes and S1 with 3206 genes for the Human PPI network. The numbers inside parenthesis after L1 and S1 represent the number of genes that DAVID could annotate, which is generally less than the number of genes in those communities. The first column lists the broad category of pathways (*M*: Metabolism, *HD*: Human Diseases, *GIP*: Genetic Information Processing, and *CP*: Cellular Processes), *FE*: Fold Enrichment

As seen in Table 11 (C1 vs. S1), there is a good overlap between enriched genes in C1 and S1 communities for different pathways. The first pathway is oxidative phosphorylation with the lowest p-value. This pathway has 102 genes enriched in C1 and 99 genes enriched in S1. Of these genes, there are 98 genes common between the two communities which is a 96% overlap. Counting all genes for all pathways yields 778 unique genes for C1 and 756 unique genes for S1. Of these genes, there are 696 genes common between the two communities which is an 89% overlap. The results of GO term enrichment analysis between these two communities are presented in Additional file 1: Table S17, where a similarity of 91% is observed.

As seen in Fig. 1, communities C1 and S1 represent six different broad categories of functions and they are similar to each other in terms of the percentage of enriched genes in each category.

Next, we will compare Louvain and Spinglass. The results of comparing the top 10 pathways for L1 and S1 are summarized in Table 12 (Additional file 1: Table S16 for the full list). Figure 2 shows the broad functional categories for comparing all pathways with *p*-values less than 0.01 for L1 and S1. Comparison of Figs. 1 and 2 reveals that L1 and S1 are less similar as compared to C1 and S1. However, it is appropriate to say that Combo, Louvain and Spinglass broadly yield similar and reasonably sized communities.

### Orthology comparison of communities from Yeast and Human PPI networks using Louvain method

In this sub-section, we will compare communities detected by Louvain for the Yeast PPI network and communities detected by the same method for the Human PPI network. Louvain could find 9 communities with sizes ranging from 4 to 1538 for the Yeast PPI network (named SC1 for the biggest and SC9 for the smallest community) and 14 communities with sizes ranging from 3 to 3585 for the Human PPI network. Using biomaRt package of R [26], we were able to find orthologous genes between Yeast and Human. Since the sizes of communities (the number of genes in the community) detected for the Human PPI network are larger than the size of communities detected for the Yeast PPI network, we found orthologous genes of the communities detected for the Human PPI network in Yeast (denoted HS ➔ SC) and then used DAVID to perform KEGG pathway enrichment for those genes. KEGG pathway enrichment results for the HS ➔ SC genes were compared to that for the communities of the Yeast PPI network. Table 13 shows the Jaccard index (as a percentage) between different pairs of communities and guided us on which community pairs should be compared with each other. For example, SC2 should be compared with HS3 ➔ SC. The results of comparing SC2 and HS3 ➔ SC are presented in Table 14. Tables for other comparisons of this sub-section are in the supplementary section (SC4 vs. HS1 ➔ SC in Additional file 1: Table S18 and SC5 vs. HS2 ➔ SC in Additional file 1: Table S19).

**Table 13:**
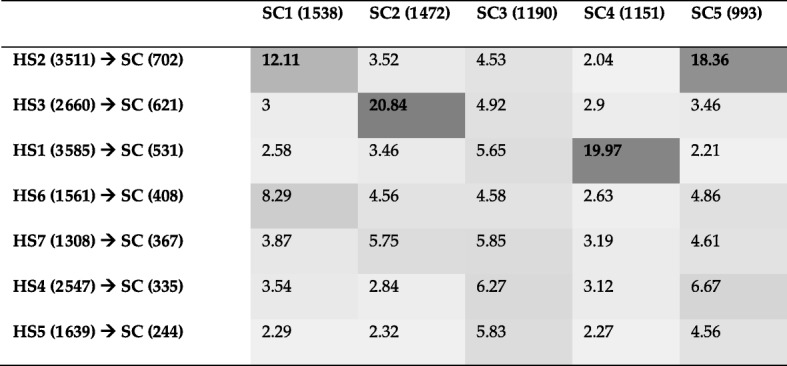
Jaccard index (as a percentage) between the communities detected by Louvain for the Yeast PPI network and orthologous genes of the communities detected by the same method for the Human PPI network in Yeast. Community pairs with maximum overlap of more than 10% (e.g., SC2 vs. HS3 ➔ SC) are indicated in bold text

**Table 14:**
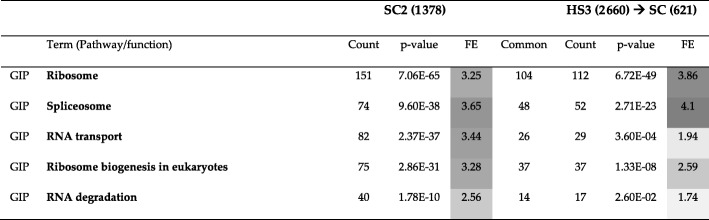
A comparison of KEGG pathway enrichment results between the second community detected by Louvain for the Yeast PPI network (SC2) and HS3 ➔ SC (orthologous genes of the third community of the Human PPI network in Yeast). The first column lists the broad category of pathways (*GIP*: Genetic Information Processing), *FE*: Fold Enrichment

As seen in Table 14, the most enriched pathway is the ribosome pathway with 151 genes enriched in SC2 and 112 genes enriched in HS3 ➔ SC. Of these, 104 genes are common between the two communities, which is a 69% overlap. Counting all genes for all pathways yields 380 unique genes for SC2 and 233 unique genes for HS3 ➔ SC. Of these genes, there are 218 genes common between the two communities, which is a 57% overlap. Although this similarity level is not impressive by itself, we did not expect much overlap between the two communities since Table 13 represents only a 21% similarity between them.

## Discussion

As mentioned in the Results section, Louvain and Spinglass are most similar to each other for the Yeast PPI network (Table 2). Louvain tries to maximize the modularity (*Q*) whereas Spinglass tries to minimize the Hamiltonian (H). However, it has been shown that there is a relation between *Q* and H as $Q=-\frac{\mathcal{H}}{2M}$ (Eq. 16 in the Methods section). Thus, minimizing H is equivalent to maximizing *Q*. Still, since they use different algorithms to optimize their objective functions, the results are not exactly the same. Combo also tries to maximize modularity but in a different way than that in Louvain, thus resulting in slightly different communities as compared to those obtained by the Louvain method.

**Fig. 3:**
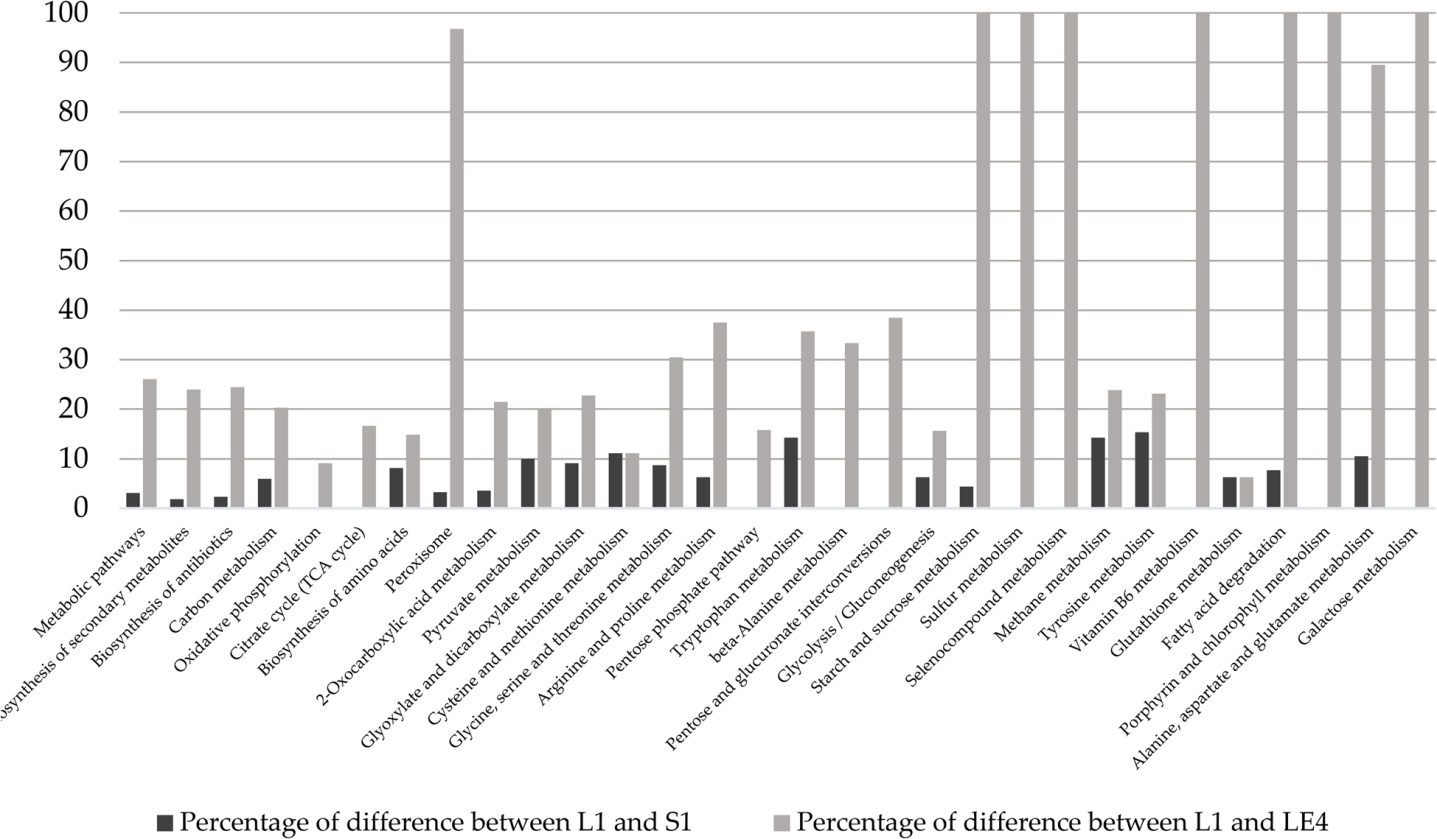
Comparing number of genes enriched in different pathways for the first community detected by Louvain (L1), the first community detected by Spinglass (S1) and the fourth community detected by Leading Eigen (LE4) for the Yeast PPI network

Table 2 suggests us that Louvain and Spinglass are most similar to each other while Louvain and Leading Eigen are most dissimilar for the Yeast PPI network. Fig. 3 illustrates the differences (as a percentage, i.e., 100*(#genes different between L1 and S1 (or L1 and LE4))/(max(L1,S1,LE4)) for each pathway) between the number of genes enriched in different pathways (with more than 10 genes and *p*-values less than 0.01) for L1 and S1 (black columns), and for L1 and LE4 (grey columns). As seen in Fig. 3, there is more difference between the number of genes enriched in L1 and LE4 compared to the difference between L1 and S1. This also verifies our results of topological comparison between L1 and S1, and L1 and LE4 (see also Table 2).

KEGG pathway enrichment results for communities detected for the Yeast PPI network show that almost all pathways of each community belong to one broad function. For example, the first community of Louvain mostly includes pathways related to metabolic processes, the second community consists of pathways related to genetic information processing. On the other hand, the functions/pathways represented by communities detected for the Human PPI network are somewhat mixed and include several broad biological functions. Vis-a-vis the functional similarity of the methods, for the Human PPI network, Combo and Spinglass are similar, e.g., Fig. 1 shows that in the first community of Combo (C1), 15% of the genes belong to pathways related to human diseases and the first community of Spinglass (S1) also has 15% of the genes related to the same broad category.

In order to confirm that the size of communities detected by different methods are reliable, we found sub-communities of the communities detected by Combo, Louvain and Spinglass for the Yeast PPI network and analyzed the results to see if detected sub-communities of one community include pathways related to different biological functions or to the same one as the main community. As an example, most pathways of L1 belong to metabolic processes and the pathways for its sub-communities also belong to metabolic processes. Due to the result of this comparison and other comparisons for all communities and sub-communities detected by Combo, Louvain and Spinglass, we can conclude that the size of communities detected are reliable.

We were also curious to know if all genes enriched in each pathway belong to one community or to different communities. To find this, we compared genes enriched in different pathways for all communities detected by Combo, Louvain and Spinglass for the Yeast PPI network. The results of this comparison are shown in Tables 15, 16 and 17. The first column lists the broad category of pathways (M: Metabolism, and GIP: Genetic Information Processing). The second column lists the different pathways enriched, columns 3 through 7 represent number of enriched genes for each community, column 8 is the summation of all enriched genes and the last column specifies total number of genes in each pathway in DAVID KEGG database. Some numbers in these tables are colored in grey, meaning their *p*-values are larger than the cut-off of 0.01. As seen in these tables, most enriched genes in each pathway belong to one community and the corresponding pathway is also significantly enriched. There are only a few exceptions such as metabolic pathways (which has a total of 685 genes and they are mainly distributed into two communities while still maintaining p-value less than 0.01 for the pathway (Tables 15-17, for the Yeast PPI network)), biosynthesis of amino acids (Table 16) and ribosome (Table 15). For the other pathways, most of the enriched genes belong to one community and if another community has some enriched genes, the p-value is greater than the cutoff of 0.01 (colored grey).

**Table 15:**
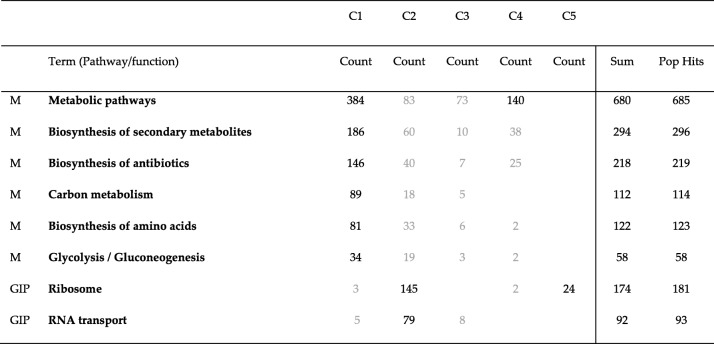
Number of genes enriched in each pathway for different communities detected by Combo for the Yeast PPI network. The first column lists the broad category of pathways (M: Metabolism, and GIP: Genetic Information Processing). Column 2 lists the different pathways enriched. Columns 3 through 7 represent number of enriched genes for different communities. Column 8 lists the total of all enriched genes of all communities together and the last column represents the maximum number of genes in that pathway. For example, in DAVID database, “metabolic pathways” contains 685 genes and of these, 384 were found in C1 and 140 were found in C4

**Table 16:**
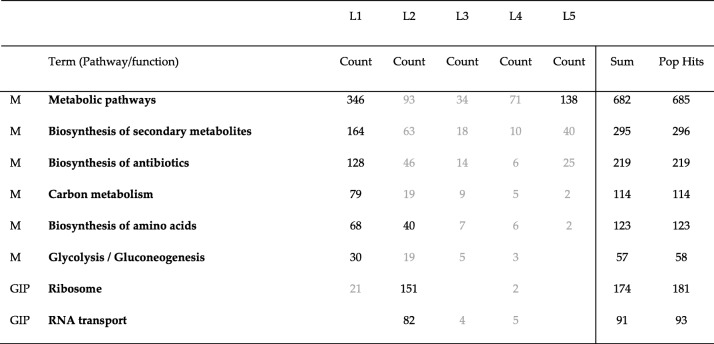
Number of genes enriched in each pathway for different communities detected by Louvain for the Yeast PPI network. This table is arranged similar to Table 15

**Table 17:**
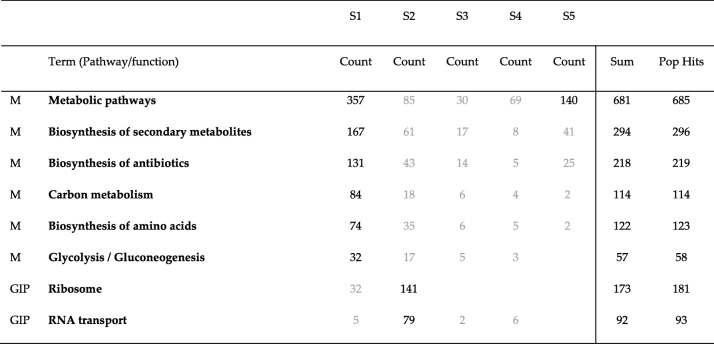
Number of genes enriched in each pathway for different communities detected by Spinglass for the Yeast PPI network. This table is arranged similar to Table 15

We note that the analysis provided above does not fully address the issue of selecting the best method. It is likely to be subjective and network specific. Hence, we recommend applying a few different methods, such as Louvain (from the group of similar methods) and one or two other (dissimilar) methods, and compare and interpret the results to obtain a consensus best method.

### Robustness of communities obtained by the Louvain method

We also analyzed the robustness of communities obtained by Louvain method for small perturbations in the network. Essentially, the network is randomly perturbed by deleting some nodes (and edges involving them) and the communities are identified. This is carried out 100 times to assess the robustness of the communities. First, the communities of the original Yeast PPI network are identified using Louvain method. Then, the following steps are repeated 100 times:

1. Remove 1% of nodes randomly (e.g., 65 nodes out of 6532 nodes for the Yeast PPI network).
2. Find the communities of the new network using Louvain method.
3. From the communities of the original network, delete the random nodes of step 1.
4. Calculate the Jaccard index matrix between the communities obtained in steps 2 and 3. We considered all communities with more than 100 nodes.
5. Compute the maximum value for each column of the Jaccard index matrix.
6. Compute the average of the resulting row-vector.

**Fig. 4:**
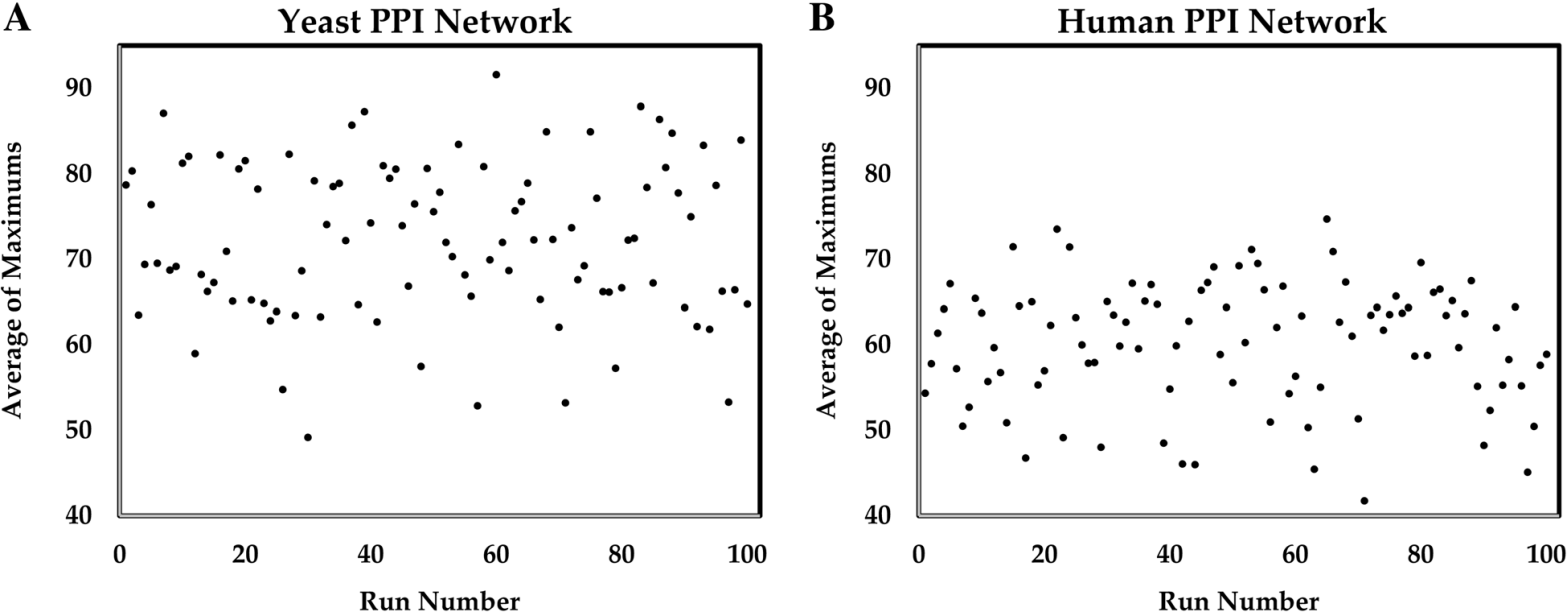
Scatter plots of avg-max (average of column-wise-maximum of Jaccard index matrix) values vs. run number. The left panel (**a**) shows the results for the Yeast PPI network and the right panel (**b**) shows the results for the Human PPI network

After performing the above steps 100 times, we have the vector of average of column-wise-maximum (avg-max) of Jaccard index values. Then, we generate scatter plots of avg-max. The scatter plots of avg-max for the Yeast and the Human PPI networks are shown in Fig. 4 (left panel: Yeast, right panel: Human).

The mean and standard deviation of the avg-max vector are 72.21 and 9.02%, respectively for the Yeast PPI network, whereas, for the Human PPI network, they are 60.11 and 7.14%, respectively. These relatively large numbers for the mean suggest that the communities identified by Louvain method are robust to small perturbations. We repeated the process for Leading Eigen. The mean and standard deviation of the avg-max vector for the Yeast network is 53.4 and 5.63%, respectively. Thus, Louvain is a better method, at least for the Yeast PPI network; for the Human PPI network, Leading Eigen finds only two or three very large communities, making avg-max artificially high (98%).

### Generality of the overall results

The PPI networks that we used in our analysis were from BioGRID and included both physical and genetic interactions (combined network). Hence, we have also applied the various approaches for finding communities to a Yeast PPI network comprising of only physical interactions, which has 6298 nodes and 83,788 edges, to find out if our broad conclusions based on the combined network are still valid for the physical interaction-only network. Additional file 1: Table S20 shows the modularity scores and the number of communities detected by different methods for the physical interaction-only network. While Q is smaller for the combined network as compared to that for the physical interaction-only network for all methods, their relative trend for the different methods remain almost the same. Interestingly, the Q values for Louvain, Combo and Spinglass are similar and among the largest. Thus, these three methods are one of the best methods in terms of Q value as well.

We have compared the various methods using three topological metrics (namely Rand Index, Adjusted Rand Index, and Normalized Mutual Information) with respect to the physical interaction-only network. The results for these comparisons are given in Additional file 1: Table S21. Based on results of Additional file 1: Table S21, Combo, Louvain and Spinglass are similar to each other in terms of the topological metrics. We also compared the KEGG pathway enrichment results for the first 5 communities of Louvain and Spinglass. To find which communities from Louvain and Spinglass methods are similar, we generated the Jaccard index matrix for communities with more than 100 nodes for both methods (Additional file 1: Table S22). After selecting pairs of communities with highest value of Jaccard index for each column, we used DAVID version 6.8, to perform KEGG pathway enrichment analysis. The results of those comparisons are presented in Additional file 1: Tables S23-S25 for the L1 vs. S1, L2 vs. S2 and L3 vs. S4 comparisons, respectively. These tables are arranged similar to Additional file 1: Table S4.

**Fig. 5:**
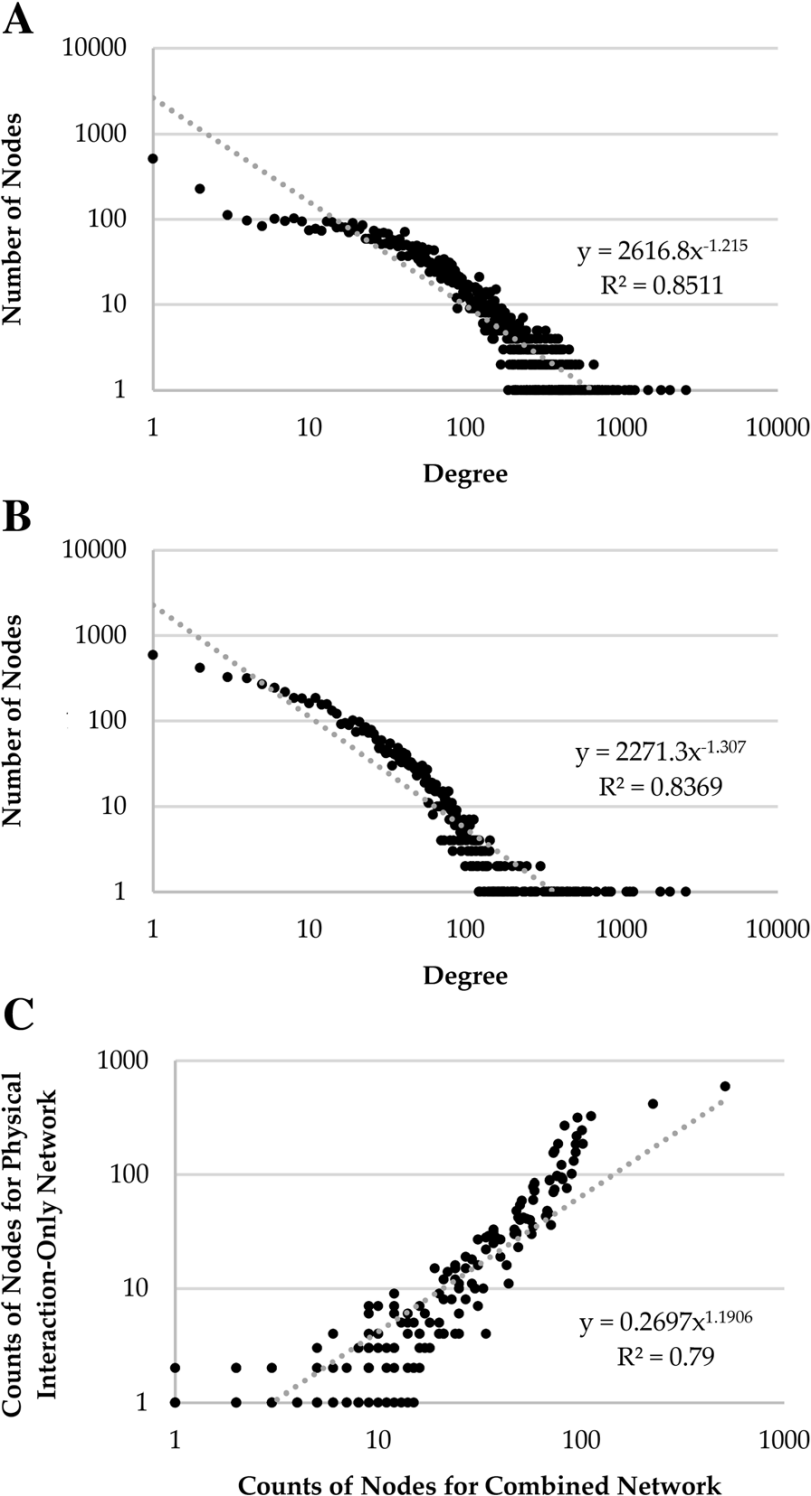
**a** Node-degree distribution for the combined network. **b** Node-degree distribution for the physical interaction-only network. **c** Comparison of counts of nodes between the combined network and the physical interaction-only network

Two main results from Additional file 1: Tables S23-S25 are: 1) good functional similarity between the communities from Louvain and Spinglass methods (e.g., an overlap of 73.06% between L1 and S1, 79.41% between L2 and S2 and 85.24% between L3 and S4), and 2) segregation of biological functions represented by the communities, e.g., communities L2, L3 and L4 represent mostly metabolism related pathways. L1 shows a mixed enrichment, akin to the mixed pathways represented by the L3 and L5 communities of the combined network (Additional file 1: Tables S4 and S6). Some differences in the nature of the broad results for the combined network vs. the physical interaction-only network are likely due to the fact that the physical interaction-only network is much sparser (just 1/3rd of the edges are retained) as compared to the combined network. Using Cytoscape [27], we also analyzed the properties (Additional file 1: Table S26) and node-degree distribution for the combined network and the physical interaction-only network (Figs. 5(a) and 5(b)). Figure 5(c) shows a comparison of count of nodes for a given degree between the combined and the physical interaction-only networks. As can be seen from Fig. 5(c), good R^2^-value suggests good agreement between the two degree distributions.

### Optimization of method-specific parameters

We wanted to find out if the Q value for the different methods could be improved by optimizing their parameters, if any, or if their results varied across different runs. Of six methods, three of them, namely Combo, Conclude and Spinglass used a random number generator in the procedure for finding communities, although they do not have any parameters to be optimized. We carried out 10 repeats of Combo, Conclude and Spinglass on the combined Yeast PPI network to assess the variation in Q across the runs. Additional file 1: Table S27 shows the results of 10 runs for these methods (similar to those for the Combo and Spinglass methods for the Human PPI network in Tables 8 and 9, respectively). The standard deviations of Q across the 10 runs are much larger for Conclude (5.11%, std./mean) as compared to those for Combo (0.05%) and Spinglass (0.36%). Thus, for a small number of allowed runs, the results from Combo and Spinglass are more reliable.

We selected the run with the highest modularity for Conclude method and performed KEGG pathway enrichment analysis. Then we compared these enrichment results with the enrichment results for the communities from the Conclude method reported earlier. Although the size of communities are a little different, the broad categories of pathways for majority of the communities are still the same. For example, there is one community in both runs which has pathways mainly related to metabolic processes. Additional file 1: Table S28 shows the results for comparison between the two communities with pathways related to metabolic processes (CL3) in the two runs.

Spinglass method uses simulated annealing in its procedure. Hence, we ran it 10 times with 10 different start and stop temperatures and cooling factor, but the default values in the igraph package yielded the best result in terms of the largest modularity. Overall, at a broad level, we found that in our case studies, the methods providing well-interpretable communities also resulted in near-optimal (largest) Q values. The fact that the Q value for Conclude in one of the runs is slightly higher (0.29) than those for Combo and Spinglass (0.265) does not violate this conclusion because some of the communities from Conclude are also well-interpretable. However, we note that the Q values across different runs vary substantially for Conclude as compared to those for Combo and Spinglass, suggesting that Combo and Spinglass methods are likely more robust than the Conclude method.

## Conclusions

In this paper, we tested six methods to detect communities within the Yeast and Human PPI networks. An in-depth comparison of communities detected by these different methods has led us to conclude that Louvain and Spinglass are most similar for the Yeast PPI network whereas Combo and Spinglass are most similar for the Human PPI network. In terms of finding communities that include core pathways based on KEGG pathway and GO term enrichment results, Combo, Louvain and Spinglass were able to find similar communities in which important biological functions and pathways were enriched. For the Yeast PPI network, all genes from the network belonging to a pathway also generally belong to only one or two communities. In terms of running time or computational complexity, for the Yeast PPI network, Louvain was the fastest method and Combo was faster than Spinglass. For the Human PPI network, Louvain was much faster than Spinglass, and Spinglass was faster than Combo. Overall, Combo, Louvain and Spinglass provide reasonable results for community detection in biological networks. Their corresponding modularity values were also among the highest, except that Conclude yielded slightly better modularity for the Yeast PPI network in some runs; variation in modularity for Conclude was much larger than that for Combo and Spinglass across multiple runs. Overall, since Combo and Spinglass use stochastic search in their procedure and their running time is also more than that for Louvain, Louvain is likely the best method to find reasonably sized communities for biological networks in a reasonable time. While we applied these methods to PPI networks, we expect the broad results to be applicable to other types of networks such as gene-coexpression networks and hybrid/integrated networks.

## Methods

The focus of this work is on undirected, unweighted and connected graphs defined as *G(n,m)* where *n* = {*n*_*1*_, *n*_*2*_, …, *n*_*|n|*_} is an ordered set of nodes and *m* a subset of n*n, is the set of edges; for convenience, here, *n* and *m* represent sets. In our case studies on the Yeast and Human PPI networks, each node represents an Entrez gene ID and each edge represents an interaction between two nodes. By finding communities, we imply the segregation of nodes into groups such that there is a higher density of edges within each group than between groups. Although there are many algorithms for detecting communities, we selected six algorithms that performed well in terms of finding core pathways in biological networks with several thousands of nodes and hundreds of thousands of edges within reasonable computing time (e.g., one day on a 24 processor machine).

Each of these algorithms tries to optimize an objective function which in most cases is modularity. However, two algorithms with the same objective function will generally yield slightly different communities using different amounts of run time if they use different optimization strategies. The algorithms that will be briefly described below are: Girvan-Newman [1, 7], Fast Greedy [11], Combo [28], Louvain [10], Conclude [29], Infomap [30], Leading Eigen [8] and Spinglass [9]. We used an R package named igraph [31] to run Fast Greedy, Louvain, Leading Eigen and Spinglass. There is a Cytoscape plugin, named GLay, to visualize the network and the communities [32]. The GLay plugin utilizes igraph C library which provides the same methods as the igraph R package [31]. For the other two methods (Conclude and Combo), authors have made java [33] and C++ [34] codes of their algorithms available online. Girvan-Newman method did not provide any result for our networks in 24 h on a 24 processor machine, so we did not consider its results in our comparison. Another method, called Infomap, did not find reasonably-sized communities (e.g., for the Yeast PPI network with 6532 nodes, one community has 6195 nodes, and the rest have less than 10 nodes). Due to these reasons, we did not consider Girvan-Newman and Infomap methods in our analysis. However, Fast Greedy and Louvain, which we have used in our comparison, are based on the original Girvan-Newman method. So, we will briefly describe Girvan-Newman algorithm in the next subsection.

### Algorithms for community detection

#### Girvan-Newman

This is a divisive algorithm for identifying communities in networks where edges are iteratively removed based on the value of their betweenness. The steps of the algorithm are as follows [1, 7]:

1. Calculate betweenness score for all edges in the network. Betweenness is a measure that favors edges that lies between communities and disfavors those that lie inside communities. While there are many measures to find betweenness, Girvan-Newman algorithm uses the fast algorithm of Newman to find betweenness scores [35].
2. Find the edge that has the maximum value of betweenness and remove it from the network.
3. Recalculate betweenness for all remaining edges in the network and repeat from step 2 for the new scores until there are no edges remaining.

The output of this algorithm is a dendrogram that captures the possible divisions of the network into communities. In order to select the optimal division among all possible options, Modularity (Q) is used.

1 $$ Q=\frac{1}{2m}\sum \limits_{i,j}\left[{A}_{ij}-\frac{k_i{k}_j}{2m}\right]\delta \left({c}_i,{c}_j\right) $$

where *A*_*ij*_ is the weight of the edge between node *i* and *j* (is equal to *1* when all edges have the same weight), *k*_*i*_ is the sum of the weights of the edges attached to node *i* or degree of node *i*, *c*_*i*_ is the community to which node *i* belongs to and the *δ* function is defined as *δ(u,v) = 1* if *u = v* and *0* otherwise. Local peaks in the modularity during the process of community detection indicate good divisions of the network into communities. While using this algorithm gives good results in many cases, its computational complexity is *O(m*^*2*^*n)* or *O(n*^*3*^*)* on a sparse graph, where *n* is the number of nodes and *m* is the number of edges. This makes it impractical for very large graphs with several thousands of nodes and hundreds of thousands of edges.

#### Fast greedy by Clauset, Newman and Moore

This algorithm (namely, Fast Greedy) involves finding the changes in modularity that results from combining pair of communities, selecting the combination yielding largest gain in modularity, and implementing the combination of the corresponding pair [11]. At the beginning, each node is considered as a community. One way of performing the combination process is to consider the network as a multigraph where a whole community is represented by a node and the elements of the adjacency matrix are equal to the number of edges between the communities. Joining two communities (namely *i* and *j*) corresponds to replacing the *i*^th^ and *j*^th^ rows and columns of the adjacency matrix by a single row and column formed by their sum, respectively; the record of the list of nodes in the communities formed thus far is updated. In the algorithm proposed by Newman [13], this operation is carried out explicitly on the entire adjacency matrix. Calculating the change of modularity (Δ*Q*) and finding the pair *i*, *j* with the largest gain is time-consuming. Hence, here, instead of using the adjacency matrix and calculating Δ*Q*_*ij*_, a matrix of Δ*Q*_*ij*_ values is initialized and updated directly. For sparse matrices, e.g., adjacency matrices for large networks, this results in substantial reduction in computation. The following parameters have to be set initially:

2 $$ {\Delta  Q}_{ij}=\left\{\begin{array}{c}\raisebox{1ex}{$1$}\!\left/ \!\raisebox{-1ex}{$2m$}\right.\kern0.35em -\kern0.35em \raisebox{1ex}{${k}_i{k}_j$}\!\left/ \!\raisebox{-1ex}{${(2m)}^2$}\right.\ \mathrm{i}\mathrm{f}\ \mathrm{i},\mathrm{j}\ \mathrm{are}\ \mathrm{connected}\ \\ {}0\ \mathrm{otherwise}\end{array}\right. $$

3 $$ {a}_i=\frac{k_i}{2m} $$

for each *i*. The next steps are as follows:

1. Calculate the initial values of Δ*Q*_*ij*_ and *a*_*i*_ based on Eqs. 2 and 3, and form the max-heap, *H*, which contains the largest element of each row of the matrix Δ*Q*_*ij*_ along with the labels *i*, *j* of the corresponding pair of communities.
2. Select the largest Δ*Q*_*ij*_ from *H*, combine the corresponding communities, update the matrix *ΔQ*, *H* and *a*_*i*_ (Eq. 4) and increase *Q* by Δ*Q*_*ij.*_
3. Repeat step 2 until only one community exists.

Due to the sparsity of the original adjacency matrix *A*, we will be able to perform updates in step 2 quickly and we need to only adjust a few elements of *ΔQ.* If communities *i* and *j* are joined together, labeling the combined community as *j*, we update the *j*^th^ row and column, and delete the *i*^th^ row and column altogether. The update rules are as follows:

If community *k* is connected to both *i* and *j*, then:

4a $$ \Delta  {Q}_{jk}^{\prime }=\Delta  {Q}_{ik}+\Delta  {Q}_{jk} $$

If *k* is connected to *i* but not to *j*, then:

4b $$ \Delta  {Q}_{jk}^{\prime }=\Delta  {Q}_{ik}-2{a}_j{a}_k $$

If *k* is connected to *j* but not to *i*, then:

4c $$ \Delta  {Q}_{jk}^{\prime }=\Delta  {Q}_{jk}-2{a}_i{a}_k $$

To update *a*: $$ {a}_j^{\prime }={a}_j+{a}_{i.} $$

Fast Greedy algorithm runs in time *O(m.d.logn)* for a network with *n* nodes and *m* edges where *d ~ logn* is the depth of the dendrogram. For sparse networks (*m ~ n*), the running time is *O(nlog*^*2*^*n)*, which is essentially linear [11].

#### Combo

Most community detection algorithms use one of the following steps in the process of finding communities: they may join two communities, split a community into two, or move nodes between two distinct communities. Combo involves all three possibilities [28]. After selecting an initial partition made of a single community, the following steps are iterated until there is no gain in the objective function which may be modularity (Eq. 1) or description code length:

1. For each source community, the best possible repartition of every source node into each destination community (either an existing community or a new community) is calculated. It would be possible that the source community totally joined the destination community in this step.
2. The best merger, split, or recombination is performed.

The basis of Combo is the selection of the best repartition of nodes between two communities. For each pair of source and (maybe empty) destination communities, a shift of all the nodes using Kernighan-Lin algorithm [36] is performed. Particularly, Combo recombines the two communities starting from several initial configurations including: (a) the original communities, (b) the case in which the whole source community is moved to the destination community and (c) a few intermediate mergers, where a random subset of the source community is shifted to the destination community.

For each starting configuration, a series of Kernighan-Lin shifts [36] is iterated until no further improvement is possible. Each configuration is carried out by initializing a list of available nodes to cover all the nodes from the original source community and then iterating the following steps until there are no more nodes in the list:

1. Find the node *i* in the list which when switched to another community results in the largest gain in modularity.
2. Switch *i* to the other community, remove it from the original list and save the intermediate result.

After performing a series of Kernighan-Lin iterations for each of the starting configurations, the intermediate result with the best score in terms of objective function (modularity) is selected.

Combo outperforms all other known algorithms when the objective function is modularity. However, it has limitations on the size of the network it could handle within a reasonable time, which is currently around 30,000 nodes and is not a serious limitation for most biological networks. When the objective function is description code length, Combo’s results are similar to Infomap (which will be described later in this section) in most cases. Since the sequence of operations depends on the specific network, obtaining exact evaluations of the computational complexity of Combo is difficult, but the upper bound is *O(n*^*2*^*logc)* where *n* is the number of nodes and *c* is the number of communities in the network [28].

#### Louvain

This algorithm detects communities in large networks by maximizing modularity and is much faster as compared to other methods [10, 37, 38]. The limiting factor for this method is the memory (RAM) requirement rather than the computation time, as is the case with Girvan-Newman and Spinglass algorithms. The algorithm is divided in two phases, which are repeated iteratively. First phase is to assign a different community to each node of the network. So, in the beginning, there are as many communities as there are nodes. Then, the gain of modularity (Eq. 5) is calculated for removing node *i* from its community and placing it in one of its neighboring communities. The gain of modularity in moving node *i* into a community *C* can be computed by:

5 $$ \Delta Q=\left[\frac{\Sigma_{in}+{k}_{i, in}}{2m}-{\left(\frac{\Sigma_{tot}+{k}_i}{2m}\right)}^2\right]-\left[\frac{\Sigma_{in}}{2m}-{\left(\frac{\Sigma_{tot}}{2m}\right)}^2-{\left(\frac{k_i}{2m}\right)}^2\right] $$

where Ʃ_in_ is the sum of the weights (or count for un-weighted networks) of the edges inside *C*, Ʃ_tot_ is the sum of the weights of the edges incident to nodes in *C*, *k*_*i*_ is the sum of the weights of the edges incident to nodes *i* (degree of *i*), *k*_*i,in*_ is the sum of the weights of the edges from *i* to nodes in *C* and *m* is the sum of the weights of all the edges in the network. If the gain is positive, the node *i* is placed in the community for which the gain is maximum. This process is applied repeatedly for all nodes until no further improvement can be achieved.

The second phase is to build a network whose nodes are now the communities detected during the first phase. In order to perform that, the weights of the edges between the new nodes are given by the sum of the edges between nodes in the corresponding two communities. Edges between nodes of the same community result in self-loops for this community in the new network. When this phase is completed, the first phase of the algorithm is reapplied to the new network. The combination of these two phases is referred to as a “pass”. The passes are iterated until a maximum of modularity is reached. This algorithm is extremely fast (*O(nlogn)*) and could be even faster by using some heuristics, e.g., stopping the first phase when the gain of modularity is less than a given threshold [10].

#### COmplex Network CLUster DEtection (CONCLUDE)

CONCLUDE is a fast community detection method. The algorithm takes a graph *G*, an integer *κ* and an integer *φ* as inputs. The steps are:

1. Compute κ-path edge centralities using Edge Random Walk κ-path Centrality (ERW-Kpath) algorithm (described by De Meo, et al. [29]) on nodes of *G* by carrying out at most *φ* iteration. The output of ERW-Kpath algorithm is an array of weights.
2. Calculate the distance among all pairs of nodes by taking two inputs: graph *G* and the array of weights from the previous step. It uses the following equation (Eq. 6) to find pairwise distances:

6 $$ {\sigma}_{ij}=\sqrt{\frac{\sum \limits_{k\epsilon N(i)- CN\left(i,j\right)}{\left[{L}^{\kappa}\left({e}_{k i}\right)\right]}^2}{\mid N(i)- CN\left(i,j\right)\mid }+\frac{\sum \limits_{k\epsilon N(j)- CN\left(i,j\right)}{\left[{L}^{\kappa}\left({e}_{k j}\right)\right]}^2}{\mid N(j)- CN\left(i,j\right)\mid }+\frac{\sum \limits_{k\epsilon CN\left(i,j\right)}{\left[{L}^{\kappa}\left({e}_{k i}\right)-{L}^{\kappa}\left({e}_{k j}\right)\right]}^2}{\mid CN\left(i,j\right)\mid }} $$

where *L*^*κ*^ is κ-path edge centrality and is calculated by:

7 $$ {L}^k(e)=\sum \limits_{s\epsilon V}\Pr \left(e,s\right) $$

*Pr(e,s)* is the probability of selecting the edge *e* in a random simple κ-path originating from an arbitrary source node *s*. The symbol *N(i)* is the set of neighbors of the node *i* and *CN(i,j)* indicates the subset of neighbors common to *i* and *j*.

The output of this step is a matrix *Δ* containing all pairs of distances between nodes.

3. Finally, apply Louvain method [10] on matrix *Δ* to find communities of *G*.

#### Maps of random walk (Infomap)

The Infomap approach closely follows the Louvain approach [10]; neighboring nodes are joined together to make small communities which subsequently are joined into bigger communities. The difference between these two methods is that the objective function of Louvain is modularity while the objective function of Infomap is a lower bound on a quantity referred to as code-length (*M*), defined as,

8 $$ L(M)={q}_{\curvearrowright }H(Q)-\sum \limits_{i=1}^c{p^i}_{\circlearrowright }H\left({P}^i\right) $$

The aim of Infomap is to minimize the lower bound, *L(M)*. The equation comprises of two terms: first is the entropy of the movement of nodes between communities and second is the entropy of movements of nodes within communities. Further details about this equation can be found elsewhere [30].

Each node is initially assigned to its own community. Then, in random sequential order, each node is placed into the neighboring community that results in the largest decrease in *L*(*M*) (Eq. 8). If no move decreases *L*(*M*), the node will stay in its original community. This procedure is repeated in a new random sequential order each time until no move could decrease *L*(*M*). In each iteration, the nodes of the new network are the communities found at the last level and the process of joining nodes into communities is repeated on the new network until *L*(*M*) cannot be reduced further. The computational complexity of Infomap is a linear function of the number of edges, i.e., *O(m)* [30, 39].

#### Leading Eigen

Leading Eigen method [8] is also based on the modularity maximization but here, the modularity is expressed in terms of the eigenvalues and eigenvectors of a matrix called the modularity matrix, B:

9 $$ {B}_{ij}={A}_{ij}-\frac{k_i{k}_j}{2m} $$

where, *A* is the adjacency matrix, *k*_*i*_ is the sum of the weights of the edges attached to node *i* (or degree of node *i*), *k*_*j*_ is the degree of node *j* and *m* is the total number of edges. The modularity matrix is a characteristic property of the network and is independent of any division of the network into communities. The procedure of finding communities of a network with this method consists of finding the eigenvector corresponding to the most positive eigenvalue of the modularity matrix, and then dividing the network into two groups based on the sign of the elements of the eigenvector. Defining an index vector, *s*, the sign of elements are:

10 $$ {s}_i=\left\{\begin{array}{c}+1\  if\ {u_i}^{(1)}\ge 0\\ {}-1\  if\ {u_i}^{(1)}<0\end{array}\right. $$

where, *u*_*i*_^*(1)*^ is the *i*^*th*^ element of *u*_*1*_ (the normalized eigenvector of the modularity matrix). The nodes with positive sign form one community and the rest of the nodes form the other community. To avoid dividing the network into only two communities, an *n* (total number of nodes) by *c* (the number of non-overlapping communities) index matrix *S* has to be defined. Each column of this matrix is an index vector of (*0,1*) elements, such that:

11 $$ {S}_{ij}=\left\{\begin{array}{c}1\kern0.50em if\ vertex\ i\  belongs\ to\ community\ j\\ {}0\  otherwise\ \end{array}\right. $$

The modularity of this division of the network is then equal to:

12 $$ Q=\sum \limits_{i,j=1}^n\sum \limits_{k=1}^c{B}_{ij}{S}_{ik}{S}_{jk}= Tr\left({S}^T BS\right) $$

This form of modularity is different from other forms in a leading multiplicative constant *1/(2 m)* but since it has no effect on the position of the maximum of the modularity, it has been omitted from the equation. Writing *B=UDU*^*T*^ where *U* = (*u*_*1*_*|u*_*2*_*| …*) is the matrix of eigenvectors of *B*, and *D* is the diagonal matrix of eigenvalues *D*_*ii*_ = *β*_*i*_*, Q* can be written as:

13 $$ Q=\sum \limits_{j=1}^n\sum \limits_{k=1}^c{\beta}_j{\left({u}_j^T{s}_k\right)}^2 $$

The aim is still maximizing the modularity *Q*, but now, there is no constraint on the number of communities, *c* [8].

#### Spinglass

In this method, the community structure of a network is described as the spin configuration that minimizes the energy of the spin glass (Hamiltonian) with respect to the spin states (the community indices) [9]. Similar to any other quality function for an assignment of nodes into communities, Hamiltonian has to follow the principle of grouping together the nodes that are linked (there is an edge between them) and keep apart the ones that are not linked. From this, four requirements have to be satisfied: a) rewarding internal edges between nodes of the same community (in the same spin state), b) penalizing missing edges (non-links) between nodes in the same community, *c*) penalizing existing edges between different communities and d) rewarding non-links between different communities. The following equation (Eq. 14) satisfies these properties:

14 $$ {\displaystyle \begin{array}{c}\mathcal{H}\left(\left\{\sigma \right\}\right)=-\sum \limits_{i\ne j}{a}_{ij}\underset{internal\ links}{\underbrace{A_{ij}\delta \left({\sigma}_i,{\sigma}_j\right)}}+\sum \limits_{i\ne j}{b}_{ij}\underset{internal\ non- links}{\underbrace{\left(1-{A}_{ij}\right)\delta \left({\sigma}_i,{\sigma}_j\right)}}\\ {}+\sum \limits_{i\ne j}{c}_{ij}\underset{external\ links}{\underbrace{A_{ij}\left(1-\delta \left({\sigma}_i,{\sigma}_j\right)\right)}}-\sum \limits_{i\ne j}{d}_{ij}\underset{external\ non- links}{\underbrace{\left(1-{A}_{ij}\right)\left(1-\delta \left({\sigma}_i,{\sigma}_j\right)\right)}}\end{array}} $$

where, *σ*_*i*_ denotes the community index of node *i* in the graph, *δ* is the Kronecker delta function and *a*_*ij*_, *b*_*ij*_, *c*_*ij*_, and *d*_*ij*_ represent the weights of the individual contributions, respectively. If links and non-links are each weighted equally, no matter they are external or internal, *a*_*ij*_ *= c*_*ij*_ and *b*_*ij*_ *= d*_*ij*_*,* then it would be enough to consider the internal links and non-links. Convenient choices of coefficients are *a*_*ij*_ *= 1 – γp*_*ij*_ and *b*_*ij*_ *= γp*_*ij*_ where *p*_*ij*_denotes the probability that a link exists between node *i* and *j*, normalized, such that $\sum \limits_{\mathrm{i}\ne \mathrm{j}}{\mathrm{p}}_{\mathrm{i}\mathrm{j}}=2\mathrm{m}$, where *m* is the total number of edges in the network. When *γ = 1*, it leads to the natural situation in which the total amount of energy that can possibly be contributed by links and non-links is equal, i.e., $\sum \limits_{\mathrm{i}\ne \mathrm{j}}{\mathrm{A}}_{\mathrm{i}\mathrm{j}}{\mathrm{a}}_{\mathrm{i}\mathrm{j}}=\sum \limits_{\mathrm{i}\ne \mathrm{j}}\left(1-{\mathrm{A}}_{\mathrm{i}\mathrm{j}}\right){\mathrm{b}}_{\mathrm{i}\mathrm{j}}$. As we are dealing with undirected, unweighted networks, our choice of weights allows us to simplify the Hamiltonian (Eq. 14):

15 $$ \mathcal{H}\left(\left\{\sigma \right\}\right)=-\sum \limits_{i\ne j}\left({A}_{ij}-\gamma {p}_{ij}\right)\delta \left({\sigma}_i,{\sigma}_j\right) $$

where, *p*_*ij*_, the probability, can be written as ${\mathrm{p}}_{\mathrm{i}\mathrm{j}}=\frac{{\mathrm{k}}_{\mathrm{i}}{\mathrm{k}}_{\mathrm{j}}}{2\mathrm{m}}$ and *k*_*i*_ and *k*_*j*_ represent degree of node *i* and degree of node *j,* respectively. Now, minimizing H gives the number of spin states (or communities) in a network. The minimization is carried out by using simulated annealing on the entire network. This method is rather fast and the computational complexity is approximately *O(n*^*3.2*^*)*. However, it cannot be used for disconnected networks as there is no guarantee that nodes from disconnected parts of the network also have different spin states and belong to different communities.

Substituting *p*_*ij*_ as $\frac{{\mathrm{k}}_{\mathrm{i}}{\mathrm{k}}_{\mathrm{j}}}{2\mathrm{m}}$ and *γ = 1* in Eq. 15, we have:

$$ \mathcal{H}\left(\left\{\sigma \right\}\right)=-\sum \limits_{i\ne j}\left({A}_{ij}-\frac{k_i{k}_j}{2m}\right)\delta \left({\sigma}_i,{\sigma}_j\right) $$

and comparing this equation with Eq. 1 (modularity) yields:

16 $$ Q=-\frac{\mathcal{H}\left(\left\{\sigma \right\}\right)}{2m} $$

It is clear from Eq. 16 that minimizing Hamiltonian is equivalent to maximizing modularity. Thus, we expect to get same results for Louvain (which maximizes modularity) and Spinglass (which minimizes Hamiltonian) when applied to our networks.

Table 18 summarizes the above methods described in this section.

**Table 18:** Summary of community detection methods

Name of Method	Equation #	Computational complexity	Reference
Girvan Newman	1	O(m^2^n)	[1, 7]
Fast Greedy	2, 3, 4	O(nlog^2^n)	[11]
Combo	1	O(n^2^logc)	[28]
Louvain	1, 5	O(nlogn)	[10]
Conclude	6, 7	O(m)	[29]
Infomap	8	O(m)	[30]
Leading Eigen	9, 10, 12	O(n^2^)	[8]
Spinglass	15	O(n^3.2^)	[9]

### Metrics for comparison of different algorithms

To compare different methods, we used three metrics, namely, Rand Index (RI), Adjusted Rand Index (ARI), and Normalized Mutual Information (NMI). We also used Jaccard Index for measuring similarity between different communities. These metrics are based on topological similarities of the communities identified and hence are relevant for biological networks; we expect that topologically similar communities will likely yield similar biological interpretations.

#### Rand index (RI)

Rand proposes a simple measure of agreement between the results of (i.e., the communities identified by) two methods *A* and *B* [40]. RI represents the fraction of node-pairs that are distributed to the communities obtained by the two methods in a similar manner. Let *n*_*11*_ be the number of pairs of nodes from a network *G* which are both in the same community detected by method *A* and are also in the same community detected by method *B.* Let *n*_*00*_ be the number of pairs of nodes from *G* which are in different communities in *A* and are also in different communities in *B*. *n*_*00*_ and *n*_*11*_ are interpreted as agreements in the classification of the nodes from a pair. Accordingly, two disagreement quantities *n*_*01*_ and *n*_*10*_ are also defined: *n*_*01*_ (*n*_*10*_) is the number of pairs of nodes from *G* which are in the same community detected by method *A* (*B*) but they are in different communities detected by method *B* (*A*). Then, Rand Index (RI) is given by [41]:

17 $$ RI\left(A,B\right)=\frac{n_{00}+{n}_{11}}{n_{00}+{n}_{11}+{n}_{01}+{n}_{10}} $$

As seen from Eq. 17, RI has a probabilistic interpretation with respect to picking a pair of nodes at random, i.e., $$ \frac{n_{00}+{n}_{11}}{\left(\begin{array}{c}N\\ {}2\end{array}\right)} $$, which is a probability of agreement (*N* is the total number of nodes). RI is not a normalized quantity, e.g., the upper bound is 1 but the lower bound is more than zero (network dependent). Due to this lack of normalization, Hubert and Arabie [42] suggested an improvement to RI as described below.

#### Adjusted Rand index (ARI)

ARI is equivalent to a normalized Rand Index. Consider a confusion matrix for methods *A* and *B* where rows correspond to the communities in *A* and columns correspond to the communities in *B*. *N*_*ij*_, the (*i*,*j*)^th^ entry in this matrix, is the number of nodes in both community *i* of method *A* and community *j* in method *B*. Denote by *N*_*i*_*.* the sum of all columns for row *i*; thus *N*_*i.*_ is the number of nodes in community *i* of method *A*. Define *N*_*.j*_ to be the sum of all rows for column *j*, i.e. *N*_*.j*_ is the number of nodes in community *j* in method *B*. The Adjusted Rand Index (ARI), is calculated from the values *N*_*ij*_ of the confusion matrix for the two methods as follows [42]:

18 $$ {\displaystyle \begin{array}{c}{t}_1=\sum \limits_{i=1}^{c_A}\left(\begin{array}{c}{N}_{i.}\\ {}2\end{array}\right);{t}_2=\sum \limits_{j=1}^{c_B}\left(\begin{array}{c}{N}_{.j}\\ {}2\end{array}\right);{t}_3=\frac{2{t}_1{t}_2}{N\left(N-1\right)}\\ {} ARI\left(A,B\right)=\frac{\sum \limits_{i=1}^{c_A}\sum \limits_{j=1}^{c_B}\left(\begin{array}{c}{N}_{ij}\\ {}2\end{array}\right)-{t}_3}{\frac{1}{2}\left({t}_1+{t}_2\right)-{t}_3}\end{array}} $$

where, *c*_*A*_ and *c*_*B*_ are the number of communities detected by methods *A* and *B*, respectively.

#### Normalized mutual information (NMI)

Another metric to calculate the similarity between two methods is Normalized Mutual Information (NMI). NMI is the normalized form of Mutual Information (MI). MI measures similarity between the results of two methods and is given by [41]:

19 $$ MI\left(A,B\right)=\sum \limits_{i=1}^{c_A}\sum \limits_{j=1}^{c_B}\frac{N_{ij}}{N}\mathit{\log}\left(\frac{N_{ij}N}{N_{i.}{N}_{.j}}\right) $$

where, *A* and *B* are the methods being compared. The terms used in this equation are the same as those used in the equation for ARI (Eq. 18). Then, NMI between methods *A* and *B* is calculated as:

20 $$ NMI\left(A,B\right)=\frac{-2\sum \limits_{i=1}^{c_A}\sum \limits_{j=1}^{c_B}\frac{N_{ij}}{N}\mathit{\log}\left(\frac{N_{ij}N}{N_{i.}{N}_{.j}}\right)}{\sum \limits_{i=1}^{c_A}{N}_{i.}\mathit{\log}\left(\frac{N_{i.}}{N}\right)+\sum \limits_{j=1}^{c_B}{N}_{.j}\mathit{\log}\left(\frac{N_{.j}}{N}\right)} $$

#### Jaccard index

Jaccard index is a measure of similarity for two sets of nodes, with a range from *0* to *1* and is defined as the size of the intersection (overlap) divided by the size of the union of the sets:

21 $$ J\left(A,B\right)=\frac{\#\left(A\cap B\right)}{\#\left(A\cup B\right)} $$

where, the numerator is the number of common elements between the sets *A* and *B* and the denominator is the number of all the elements in *A* and *B* combined.

### Overall approach for topological and functional comparison of communities detected by different algorithms

**Fig. 6:**
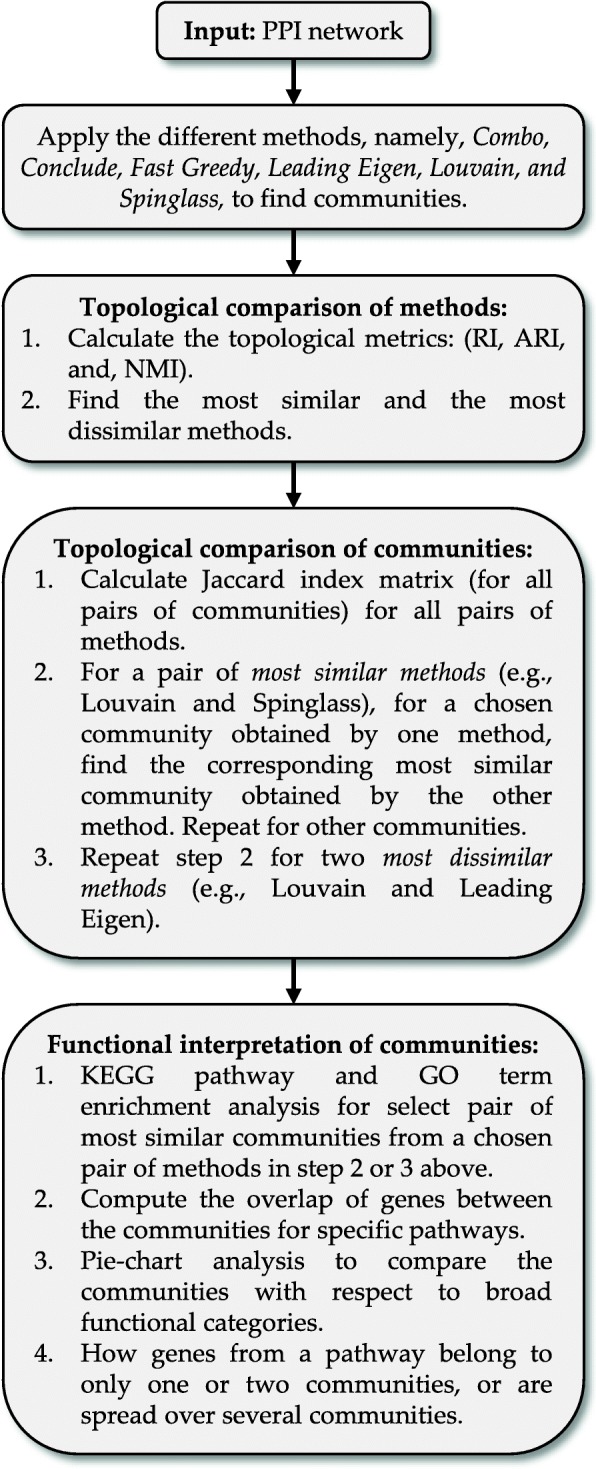
Flow chart of the steps used in our analysis

Applying the above methods to PPI networks yields different number of communities with different number of nodes. The communities detected are compared in two ways: topological comparison and functional comparison. In topological comparison, methods are compared using different metrics (RI, ARI and NMI). Based on the results of these metrics, we are able to figure out which methods are similar to each other and which are dissimilar. When a method is compared with itself, RI, ARI and NMI are 1. Larger (smaller) the value of metrics, more (less) similar are the two methods being compared. After finding which methods are similar to each other from a topological perspective, functional comparisons (such as KEGG pathway enrichment analysis) have been used to further assess the functional similarity of the communities identified by these methods. Figure 6 shows a flow chart of our analysis pipeline.

## Additional file

Additional file 1: Supplemental Tables S1-S31. (XLSX 7912 kb)

## Abbreviations

ARI: Adjusted Rand Index
CP: Cellular Processes
DAVID: Database for Annotation, Visualization and Integrated Discovery
EO: Extremal Optimization
ERW: Edge Random Walk
FDR: False Discovery Rate
FE: Fold Enrichment
GIP: Genetic Information Processing
GO: Gene Ontology
HD: Human Diseases
KEGG: Kyoto Encyclopedia of Genes and Genomes
LFR: Lancichinetti, Fortunato, Radicchi
M: Metabolism
MI: Mutual Information
NMI: Normalized Mutual Information
OS: Organismal Systems
PPI: Protein-Protein Interactions
RI: Rand Index
